# *Mettl14*-driven senescence-associated secretory phenotype facilitates somatic cell reprogramming

**DOI:** 10.1016/j.stemcr.2022.06.012

**Published:** 2022-08-09

**Authors:** Chenxiang Xi, Jiatong Sun, Xiaocui Xu, You Wu, Xiaochen Kou, Yanhong Zhao, Jiacheng Shen, Yu Dong, Kang Chen, Zhongqu Su, Dan Liu, Wen Ye, Yingdong Liu, Ran Zhang, Yiliang Xu, Hong Wang, Lujiang Hao, Li Wu, Shaorong Gao

**Affiliations:** 1 School of Bioengineering, Qilu University of Technology (Shandong Academy of Sciences), Jinan 250353, China; 2 Shanghai Key Laboratory of Maternal Fetal Medicine, Clinical and Translational Research Center of Shanghai First Maternity and Infant Hospital, School of Life Sciences and Technology, Tongji University, Shanghai 200092, China; 3 Frontier Science Center for Stem Cell Research, School of Life Sciences and Technology, Tongji University, Shanghai 200092, China; 4 Anhui Toneker Biotechnology Co., Ltd., Jinzhai, Anhui 201615, China

**Keywords:** Mettl14, reprogramming, m^6^A methylation, senescence-associated secretory phenotype (SASP)

## Abstract

The METTL3-METTL14 complex, the “writer” of N^6^-methyladenosine (m^6^A), plays an important role in many biological processes. Previous studies have shown that *Mettl3* overexpression can increase the level of m^6^A and promote somatic cell reprogramming. Here, we demonstrate that *Mettl14*, another component of the methyltransferase complex, can significantly enhance the generation of induced pluripotent stem cells (iPSCs) in an m^6^A-independent manner. In cooperation with *Oct4*, *Sox2*, *Klf4*, and *c-Myc*, overexpressed *Mettl14* transiently promoted senescence-associated secretory phenotype (SASP) gene expression in non-reprogrammed cells in the late stage of reprogramming. Subsequently, we demonstrated that interleukin-6 (IL-6), a component of the SASP, significantly enhanced somatic cell reprogramming. In contrast, blocking the SASP using a senolytic agent or a nuclear factor κB (NF-κB) inhibitor impaired the effect of *Mettl14* on reprogramming. Our results highlight the m^6^A-independent function of *Mettl14* in reprogramming and provide new insight into the interplay between senescence and reprogramming *in vitro*.

## Highlights

• *Mettl14* can facilitate somatic cell reprogramming in an m^6^A-independent manner
• *Mettl14* transcriptionally drives the senescence-associated secretory phenotype (SASP)
• *Mettl14*-driven SASPs are mainly secreted from non-reprogramming cells
• Blocking of SASP impairs the effect of *Mettl14* on reprogramming

## Graphical abstract

**Figure:**
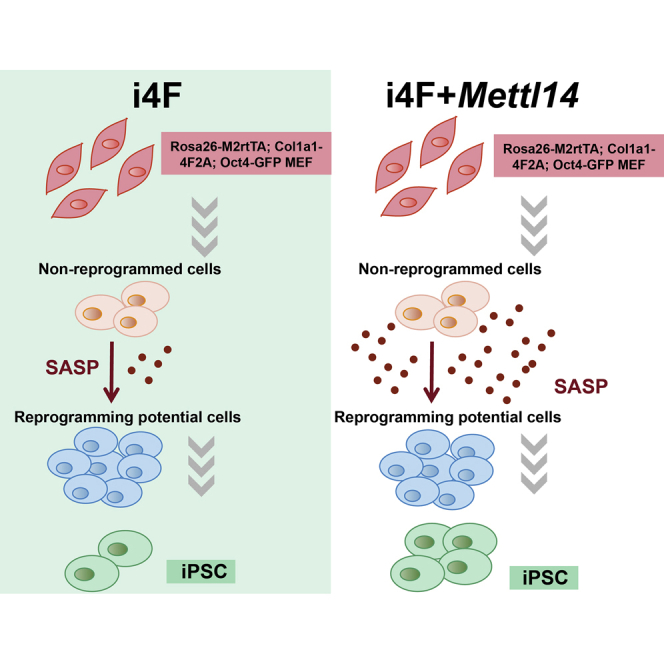

## Teaser

Gao and colleagues found that *Mettl14* can enhance somatic cell reprogramming to iPSCs in an m^6^A-independent manner. At the late stage of reprogramming, *Mettl14* significantly increased the expression level of senescence-associated secretory phenotype (SASP). These SASPs are mainly secreted by the non-reprogramming cells and blocking of SASP impairs the effect of *Mettl14* on reprogramming.

## Introduction

The N^6^-methyladenosine (m^6^A) modification is linked to human diseases because it affects multiple biological processes, including the cell cycle, fate determination, and homeostasis (Batista et al., 2014; Geula et al., 2015; Wang et al., 2014; Wen et al., 2018). Three different classes of protein factors are involved in the function of m^6^A modification: writers (adenosine MTases), erasers (m^6^A-demethylating enzymes), and readers (m^6^A-binding proteins) (Zhao et al., 2020). Deposition of m^6^A is catalyzed by the METTL3-METTL14 methyltransferase complex (MTC), and removal of m^6^A mainly depends on alpha-ketoglutarate-dependent dioxygenase AlkB homolog 5 (ALKBH5) and fat mass and obesity-associated protein (FTO) (Zaccara et al., 2019). In the m^6^A MTC complex, METTL3 mainly serves as the catalytic core, while METTL14 serves as the RNA-binding platform (Wang et al., 2016).

Reprogramming of somatic cells into induced pluripotent stem cells (iPSCs) by Yamanaka factors (*Oct4*, *Sox2*, *Klf4*, and *c-Myc*, known as OSKM) provides a system to study the molecular mechanisms of the cell-fate transition (Kang and Gao, 2012; Takahashi and Yamanaka, 2006). The role of m^6^A modifications in the generation of iPSCs is controversial, which may be due to the intricate biological functions of m^6^A (Aguilo et al., 2015; Chen et al., 2015). Increased m^6^A deposition by modulated METTL3 promotes cell reprogramming into pluripotent cells (Chen et al., 2015), but in conjunction with ZFP217 expression, downregulated METTL3 expression also contributes to reprogramming (Aguilo et al., 2015). It remains unclear how m^6^A modulates reprogramming and whether other factors of MTC affect reprogramming or whether other mechanisms are involved.

During reprogramming, accumulated damaged DNA and abnormal DNA replication cause cellular senescence. A notable signature of senescent cells is increased expression of cell-cycle-inhibitory proteins, such as p16^*Ink4a*^ and p21^*Cdkn1a*^ (Alcorta et al., 1996). In addition, senescent cells exhibit noncellular autonomous activities, such as secretion of inflammatory cytokines and chemokines (Acosta et al., 2013), which are together defined as the senescence-associated secretory phenotype (SASP) (Lopes-Paciencia et al., 2019). Transient expression of the SASP facilitates proper tissue development, tissue repair, and immune cell recruitment, but its persistent expression may induce chronic inflammation and lead to diseases associated with aging (Fitzner et al., 2012; Krizhanovsky et al., 2008; Yun et al., 2015). In senescent cells, SASP-mediated immune clearance depends on METTL14 in an m^6^A-independent manner (Liu et al., 2021).

The effect of senescence on reprogramming is still unclear. In an *in vivo* reprogramming system, induced Yamanaka factors drive cellular senescence and SASP production, which can effectively promote reprogramming (Mosteiro et al., 2016, Mosteiro et al., 2018). In addition, the most prominent cytokine in the SASP, interleukin-6 (IL-6), enhances iPSC generation, serving as an extrinsic replacement for stably transduced transcription factors such as the potent oncogene *c-Myc* (Brady et al., 2013). In this study, we identified *Mettl14* as a strong activator of *in vitro* reprogramming via transient upregulation of SASP genes in an m^6^A-independent manner.

## Results

### *Mettl14* can facilitate reprogramming in an m^6^A-independent manner

**Figure 1:**
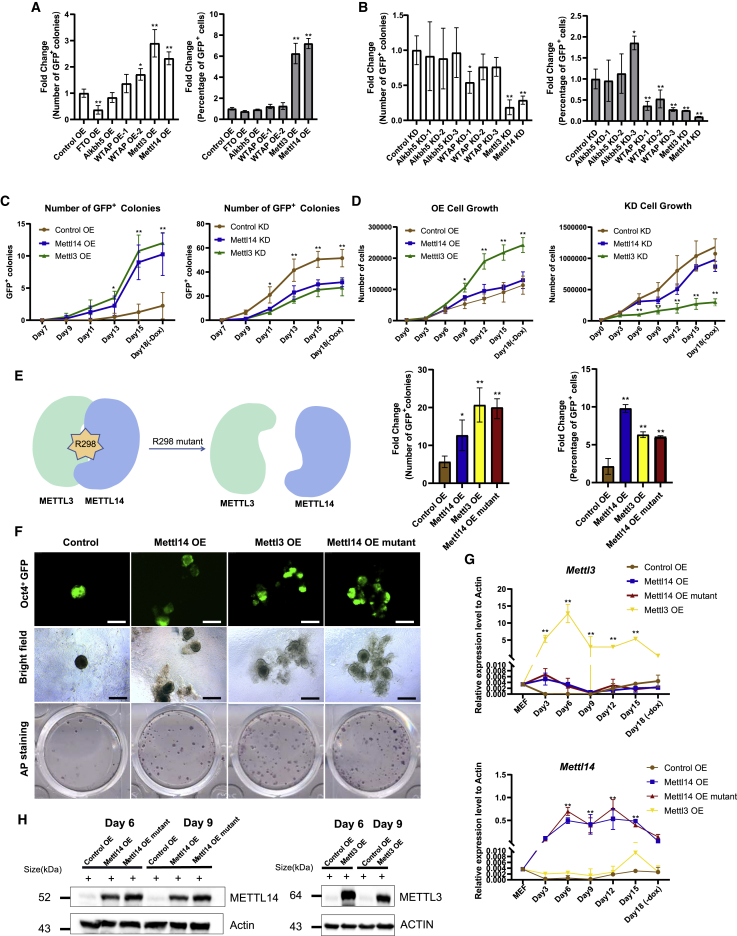
*Mettl14* can facilitate reprogramming in an m^6^A-independent manner (A) The number of *Oct4*-GFP^+^ colonies was counted, and the percentage of *Oct4*-GFP^+^ cells in the overexpression (OE) group was analyzed by FACS 18 days after induction (starting MEF density was 8,000 cells/well in a 12-well plate). The data are presented as average fold change of *Oct4*-GFP^+^ colonies (left panel) or percentage of *Oct4*-GFP^+^ cells (right panel) ± SEM (n = 3); ^∗^p < 0.05, ^∗∗^p < 0.01 by Student’s t test performed for comparison (control OE, empty vector control). (B) The number of *Oct4*-GFP^+^ colonies was counted, and the percentage of *Oct4*-GFP^+^ cells in the knockdown (KD) group was analyzed by FACS 18 days after induction (the MEF starting density was 12,000 cells/well of a 12-well plate). The data are presented as average fold change of *Oct4*-GFP^+^ colonies (left panel) or percentage of *Oct4*-GFP^+^ cells (right panel) ± SEM (n = 3); ^∗^p < 0.05, ^∗∗^p < 0.01 by Student’s t test performed for comparison (control KD, scramble short hairpin RNA [shRNA] control). (C) The number of *Oct4*-GFP^+^ colonies formed was facilitated by *Mettl14* or *Metttl3* OE. The opposite effect was observed after the expression of each was knocked down. The MEF starting density was 8,000 cells/well for OE and 12,000 cells/well for KD in a 12-well plate. The data are presented as the means ± SEM (n = 3); ^∗^p < 0.05, ^∗∗^p < 0.01 by Student’s t test performed for comparison. (D) Cells were counted at different time points during reprogramming, and growth curves were plotted. The data are presented as the means ± SEM (n = 3); ^∗^p < 0.05, ^∗∗^p < 0.01 by Student’s t test performed for comparison. (E) Schematic representation of the mutation at the *Mettl14* R298E locus (left panel). Estimated reprogramming efficiency of R298E mutant-expression cells as determined by the number of *Oct4*-GFP^+^ colonies formed and the percentage of *Oct4*-GFP^+^ cells (middle and right panels) (the MEF starting density was 6,000 cells/well in a 12-well plate). The data are presented as the means ± SEM (n = 3); ^∗^p < 0.05, ^∗∗^p < 0.01 by Student’s t test performed for comparison. (F) Morphology of the *Oct4*-GFP^+^ primary colonies (top and middle panels). Representative image of AP-stained plates captured 18 days after induction (bottom panel). Scale bars, 400 μm. (G) qRT-PCR analysis showing the expression level of *Mettl3* and *Mettl14* in the iPSCs at RNA levels. The data are presented as the means ± SEM (n = 3); ^∗^p < 0.05, ^∗∗^p < 0.01 by Student’s t test performed for comparison. (H) Western blot showing the expression level of *Mettl3* and *Mettl14* in the iPSCs at protein levels. ACTIN is used as loading control.

To investigate the effect of m^6^A level on the reprogramming process, we screened the m^6^A writers and erasers using reprogrammable mouse embryonic fibroblasts (MEFs) from *Rosa26*-M2rtTA; *Col1a1*-4F2A; *Oct4*-GFP^+^ transgenic mice (Carey et al., 2010). Exogenous doxycycline (Dox) can induce the expression of OSKM and reprogram MEFs into *Oct4*-GFP^+^ iPSCs, as previously reported (Wu et al., 2017, Wu et al., 2021). We found that *Mettl3* and *Mettl14* expression significantly increased the number of *Oct4*-GFP^+^ colonies and the percentage of *Oct4*-GFP^+^ cells (Figures 1A and S1B), while knockdown (KD) of MTC component expression remarkably reduced the number of colonies and cells (Figures 1B and S1B). Compared with the control group (overexpressing an empty vector), *Mettl14* or *Mettl3* overexpression (OE) accelerated *Oct4*-GFP^+^ colony formation and led to an approximately 6-fold increase in the number of *Oct4*-GFP^+^ colonies (Figure 1C, left panel), but the increase in *Oct4*-GFP^+^ colonies was delayed by *Mettl14* or *Mettl3* expression KD, and ultimately, the number of *Oct4*-GFP^+^ colonies was reduced (Figure 1C, right panel). To further investigate the impact of *Mettl14* and *Mettl3* on the reprogramming process, we monitored intermediate population progression. Neither *Mettl14* nor *Mettl3* affected the THY1^+^ population transition to the THY1^−^ population (Figure S1A, left panel), while the SSEA1^+^ population was significantly increased by *Mettl14* or *Mettl3* (Figure S1A, right panel). These findings suggested that *Mettl14* and *Mettl3* are involved in cell acquisition of pluripotency during reprogramming.

Although the ability to promote reprogramming was comparable, the effects of *Mettl14* or *Mettl3* on the proliferation of reprogramming cells were very different. *Mettl3* significantly accelerated cell proliferation, but *Mettl14* negligibly affected cell proliferation, during reprogramming (Figure 1D).

To further examine whether the effects of *Mettl14* are dependent on the m^6^A modification, we induced the expression of the *Mettl14* R298E mutant, which did not bind adequately with METTL3 and resulted in disruption of MTC activity (Figure 1E) (Wang et al., 2016). The *Mettl14* R298E mutant also led an increase in the number of *Oct4*-GFP^+^ colonies and percentage of *Oct4*-GFP^+^ cells (Figure 1E, middle and right panels), as well as alkaline phosphatase-positive (AP^+^) colonies (Figure 1F, bottom panel).The OE levels of *Mettl3* and *Mettl14* during reprogramming were detected at the RNA (Figure 1G) and protein levels (Figure 1H), respectively, and the expression levels of *Mettl3* and *Mettl14* were significantly increased compared with the control group.

### iPSC lines with OSKM + *Mettl14* OE exhibit pluripotency

**Figure 2:**
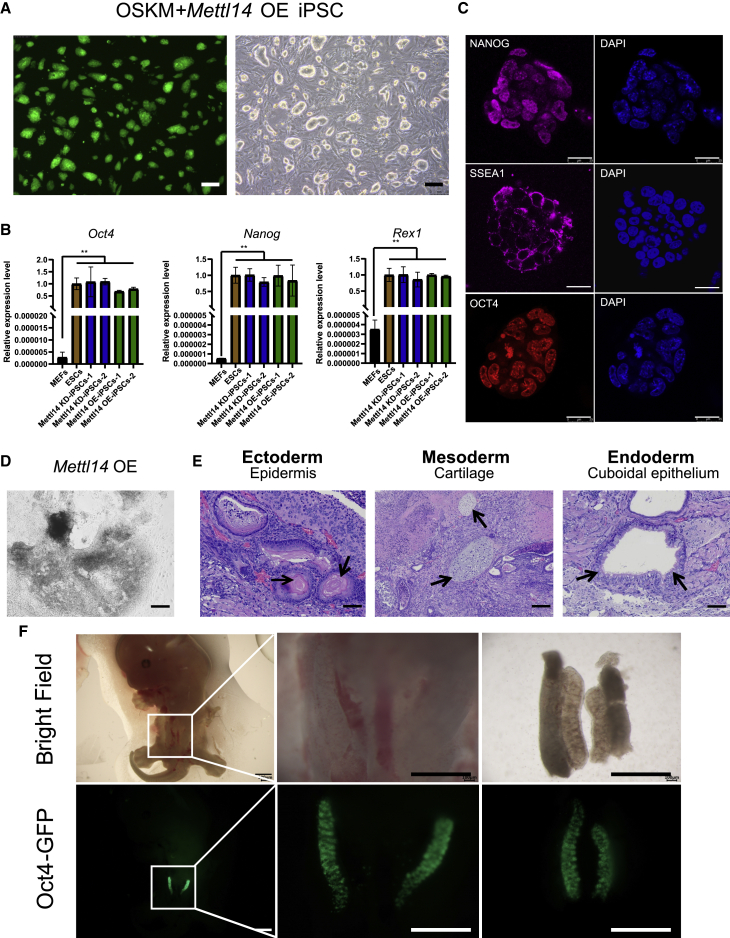
iPSC lines with OSKM+*Mettl14* OE exhibit pluripotency (A) Morphology of the iPSCs with OSKM+*Mettl14* OE lines. Scale bars, 200 μm. (B) qRT-PCR analysis showing pluripotent gene expression in the iPSCs with OSKM+*Mettl14* OE/KD relative to their expression in MEFs and ESCs. The data are presented as the means ± SEM (n = 3); ^∗^p < 0.05, ^∗∗^p < 0.01 by Student’s t test performed for comparison. (C) Immunostaining analyses for the expression of pluripotent marker genes NANOG (purple), SSEA1 (purple), and OCT4 (red) and in the iPSCs with OSKM+*Mettl14* OE lines. Nuclear staining by DAPI (blue). Scale bars, 25 μm. (D) Differentiation of the embryoid bodies of the iPSCs with OSKM+*Mettl14* OE line showing the differentiation potential. Scale bars, 200 μm. (E) Hematoxylin and eosin (H&E) staining of teratomas generated by the iPSCs with OSKM+*Mettl14* OE. Scale bars, 100 μm. (F) Representative photos showing the contribution and spatial distribution of *Oct4*-GFP^+^ cells in the gonads of the iPSCs with OSKM+*Mettl14* OE-derived chimeric embryos on embryonic day 12.5 (E12.5). Scale bars, 1 mm.

Established iPSC lines derived upon OE of *Mettl14* (OSKM+*Mettl14* OE iPSCs) exhibited typical embryonic stem cell (ESC) morphology with large nuclei and nucleoli, a compact appearance, and clear boundaries (Figure 2A). Quantitative reverse transcription PCR (qRT-PCR) showed that iPSCs with OSKM+*Mettl14* OE were comparable to ESCs in terms of mRNA expression levels of pluripotency genes such as *Oct4*, *Nanog*, and *Rex1* (Figure 2B), and protein expression levels of pluripotent genes, as shown by immunofluorescence staining (Figure 2C) (Kang et al., 2009).

To further demonstrate the quality of the iPSCs with OSKM+*Mettl14* OE, we performed *in vitro* and *in vivo* differentiation assays to detect their differentiation potential (Kang et al., 2009). Through embryoid body (EB)-mediated *in vitro* differentiation, the markers of the three germ layers in differentiated cells were significantly upregulated (Figures 2D and S2A). After subcutaneous injection of iPSCs with OSKM+*Mettl14* OE in nude mice, teratomas formed within the three germ layer tissues, which consisted of skin epithelium (ectoderm), cartilage (mesoderm), and cuboidal epithelium (endoderm) (Figure 2E) (Le et al., 2014). Furthermore, the iPSC lines with OSKM+*Mettl14* OE were successfully integrated into the gonads of chimeric mice, as shown by chimera formation assay (Figure 2F).

The iPSC lines derived from *Mettl14-*KD cells (OSKM+*Mettl14*-KD iPSCs) also exhibited an ESC-like morphology (Figure S2B), expressed pluripotent genes (Figure S2C), and differentiated into three germ layers in the teratoma assay (Figure S2D).

### Increased expression level of SASP genes after *Mettl14* OE

**Figure 3:**
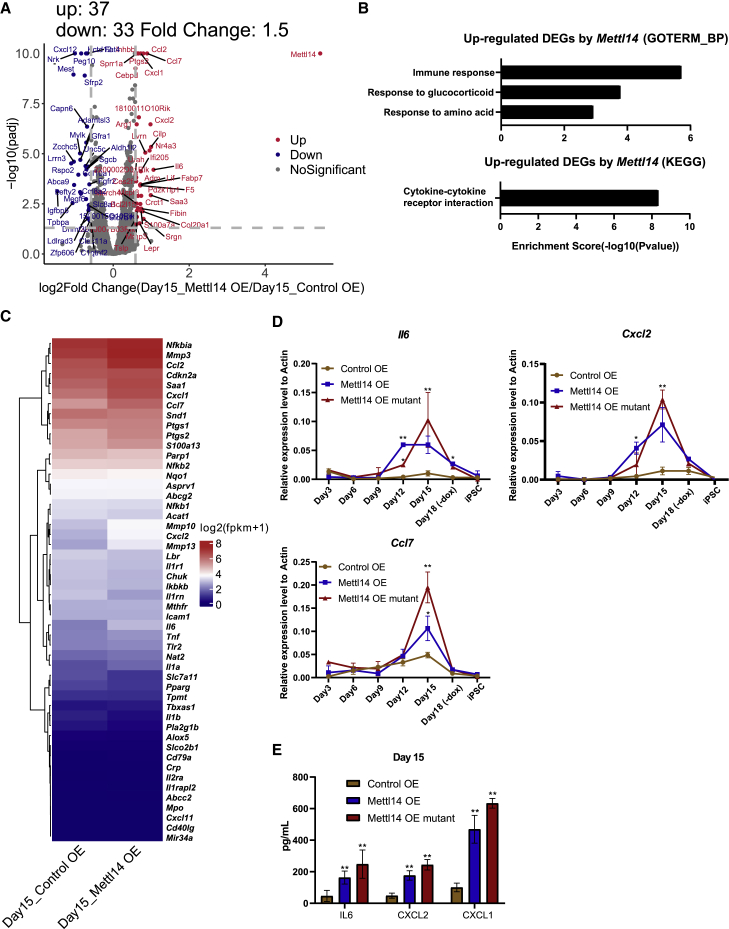
The expression level of SASP genes was increased after OE of *Mettl14* (A) Volcano plot showing the DEGs representing genetic changes caused by *Mettl14* OE on day 15 of reprogramming. (B) Gene Ontology (GO) enrichment analysis showing that the DEGs increased by *Mettl14* were mainly enriched in the immune response. (C) Heatmap showing SASP gene clustering in the samples on reprogramming day 15. (D) qRT-PCR was performed to determine the expression levels of SASP genes in wild-type and mutant *Mettl14* cells from days 3 to 18 and in iPSCs. The data are presented as the means ± SEM (n = 3); ^∗^p < 0.05, ^∗∗^p < 0.01 by Student’s t test performed for comparison. (E) ELISAs showing the expression levels of IL-6, CXCL2, and CXCL1 in the cell-conditioned medium on reprogramming day 15. The data are presented as the means ± SEM (n = 3); ^∗^p < 0.05, ^∗∗^p < 0.01 by Student’s t test performed for comparison.

To investigate how *Mettl14* facilitates reprogramming, we collected samples with or without *Mettl14* OE at various time points during reprogramming and performed RNA sequencing (RNA-seq). We performed a principal-component analysis (PCA) to compare the transcriptomes of the reprogramming cells at the indicated time points. The PC1 axis was dominated by differences among reprogramming intermediate cells. Specifically, the cells showed clear stepwise transcriptome changes during the reprogramming of MEFs (Figure S3A). Volcano plots showed that exogenous *Mettl14* treatment resulted in the upregulation of 37 differentially expressed genes (DEGs) (fold change [FC] > 1.5, false discovery rate [FDR] < 0.05) and downregulation of 33 DEGs on day 15, compared with the control group (Figure 3A).

Gene Ontology (GO) enrichment analysis showed that the DEGs increased by *Mettl14* OE were mainly enriched in immune response and cytokine-cytokine receptor interactions (Figure 3B). To understand these data, we searched the literature about immunity in cell reprogramming and *Mettl14*-related phenotypes. It has been reported that, in senescent cells, m^6^A-independent genome-wide *Mettl3* and *Mettl14* redistribution drives the SASP (Liu et al., 2021). Therefore, we analyzed the reported SASP genes (Andriani et al., 2016; Marcheggiani et al., 2021; Mosteiro et al., 2016; Suvakov et al., 2019; You et al., 2019) in our data and found that a number of SASP genes was upregulated upon treatment with exogenous *Mettl14*, as shown in the related heatmap (Figure 3C). Surprisingly, we also found that the upregulated DEGs were significantly enriched for SASP genes (7 of 37 upregulated DEGs are SASP genes, Fisher’s exact p value < 2.573e-10; Figure 3A).

To investigate how SASP genes are regulated during reprogramming, we plotted their dynamic expression levels. The expression levels of SASP genes, such as *Il6*, C-X-C motif chemokine ligand 2 (*Cxcl2*), and C-C motif chemokine ligand 7 (*Ccl7*), were increased after day 12 and peaked on day 15 (Figure S3B). To confirm the effects of *Mettl14* on late-phase reprogramming, we performed qRT-PCR assays to ascertain the expression levels of SASP genes in cells’ expression of wild-type or mutant *Mettl14* from days 3 to 18 and in iPSCs. As shown in Figure 3D, the expression levels of SASP genes peaked on day 15 and then dramatically decreased on day 18. These SASP genes were negligibly expressed or not expressed even in the established iPSC line cells (Figure 3D).

To confirm that SASP factors were secreted, we performed ELISAs to examine the secreted protein levels of IL-6, CXCL2, and CXCL1 in the late reprogramming period. The level of these factors in the medium of cells expressing either *Mettl14* wild-type or the mutant were significantly higher than those in the control group on day 15 (Figure 3E). In general, *Mettl14* transiently upregulated the expression levels of SASP genes in the late phase of reprogramming in an m^6^A-independent manner.

### SASP genes are key factors in regulating reprogramming efficiency

**Figure 4:**
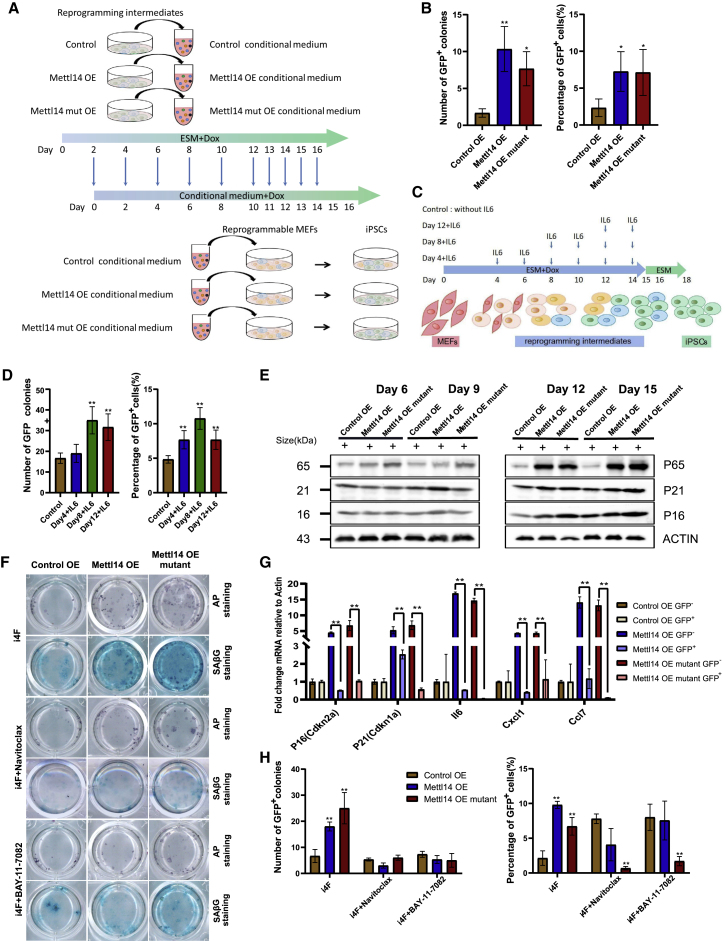
Reprogramming efficiency was reduced after SASP factors or senescence inhibitors were added to the culture (A) Schematic diagram for the procedure of collection of conditional medium from the reprogramming intermediates of different groups and treatment of reprogrammable cells. (B) Estimated reprogramming efficiency of conditioned medium from the reprogramming intermediates of different group treatments tested by the number of *Oct4*-GFP^+^ colonies formed and the percentage of *Oct4*-GFP^+^ cells. The data are presented as the means ± SEM (n = 3); ^∗^p < 0.05, ^∗∗^p < 0.01 by Student’s t test performed for comparison. (C) Schematic diagram of IL6 treatment at different time points after the induction during reprogramming. (D) Estimated reprogramming efficiency of IL6 treatment tested by the number of *Oct4*-GFP^+^ colonies formed and the percentage of *Oct4*-GFP^+^ cells. The data are presented as the means ± SEM (n = 3); ^∗^p < 0.05, ^∗∗^p < 0.01 by Student’s t test performed for comparison. (E) Western blot showing the expression level of NF-κB complexes regulatory subunit P65 and cyclin-dependent kinase inhibitors P16 and P21 during reprogramming. ACTIN is used as loading control. (F) Alkaline phosphatase (AP) staining and *β*-galactosidase staining showing changes in the number of senescent cells and GFP^+^ colonies during reprogramming before and after the treatment of Navitoclax and BAY 11-7082. (G) qRT-PCR analysis results showing the SASP gene expression levels in *Oct4*-GFP^−^ and *Oct4*-GFP^+^ cells. The data are presented as the means ± SEM (n = 3); ^∗^p < 0.05, ^∗∗^p < 0.01 by Student’s t test performed for comparison. (H) The number of *Oct4*-GFP^+^ colonies was counted, and the percentage of *Oct4*-GFP^+^ cells was determined by FACS 18 days after induction. Navitoclax and BAY 11-7082 were added to different experimental groups. The data are presented as the means ± SEM (n = 3); ^∗^p < 0.05, ^∗∗^p < 0.01 by Student’s t test performed for comparison.

Considering these findings, we hypothesized that SASP factors were secreted from intermediate cells during the phase of reprogramming. We collected the conditioned medium of the late reprogrammed cells and used it to culture untransfected reprogrammable MEFs (Figure 4A). Compared with medium used to culture the control group cells, conditioned medium obtained from *Mettl14* wild-type or mutant cell culture led to more untransfected reprogrammable MEFs transitioning into iPSCs (Figure 4B). Furthermore, to identify the SASP factors that facilitated the transition of somatic cells to iPSCs, we evaluated the effect of IL-6, a cytokine in the SASP, at different time points in the reprogramming process (Figure 4C). In the middle and late stages of reprogramming (days 8 and 12), IL-6 treatment significantly improved reprogramming efficiency (Figure 4D). These results suggested that SASP factors were secreted into the medium and regulated reprogramming efficiency.

It is thought that SASP factors are secreted mainly by senescent cells. To determine whether reprogrammed cells undergo senescence, we evaluated the protein expression levels of senescence markers P21 and P16 and that of components of the nuclear factor κB (NF-κB) pathway, which is upstream of the SASP factors. A western blot analysis showed that OE of the *Mettl14* or the *Mettl14* mutant resulted in significant upregulation in the expression level of P65, a major component of NF-κB complexes, as well as cyclin-dependent kinase inhibitors P16 and P21 during the late stage of reprogramming (Figure 4E).

To explore the possible relationship between *in vitro* reprogramming and senescence, we examined whether both of these processes proceeded within the same time period in different cell populations. We performed double staining for AP (indicating pluripotent colonies) and SAβG (indicating senescent cells) on day 15 of reprogramming (Mosteiro et al., 2016). We found a positive correlation between the degree of cell senescence and the number of AP^+^ colonies (Figure 4F). During reprogramming, wild-type *Mettl14* and mutant *Mettl14* triggered more cells to undergo senescence and generated more iPSCs (Figure 4F).

To determine which subpopulation of cells exhibited senescence and expressed SASP genes, we sorted *Oct4*-GFP^+^ and *Oct4*-GFP^−^ cells by fluorescence-activated cell sorting (FACS) on day 15 of reprogramming and measured the expression levels of senescence and SASP genes. The expression levels of senescence genes, such as *p16* (*Cdkn2a*) and *p21* (*Cdkn1a*), and of SASP genes, including *Il6*, *Cxcl1*, and *Ccl7*, in *Oct4*-GFP^−^ cells were much higher than those in *Oct4*-GFP^+^ cells (Figure 4G), suggesting that the cells that had not been successfully reprogrammed (also termed non-reprogrammed [NR] cells; Guo et al., 2019) underwent senescence and secreted SASP factors.

To track which cell population expressed SASP genes, we analyzed publicly available single-cell RNA-seq data on the cell-fate continuum during somatic cell reprogramming (Guo et al., 2019). The expression patterns of *Il6*, *Cxcl1*, and *Ccl2* were consistent with those of the NR branch signature genes (*Cd34* and *klk10*) (Figure S4A) but were very different from those of reprogramming potential (RP) branch signature genes (*Sal4* and *Dppa5a*) (Figure S4A). This result suggested that SASP-producing cells were mainly in the NR branch fraction. In addition, the expression levels of SASP genes, such as *Ccl2* and *Ccl7*, in RP cells were significantly lower than those in NR cells (Figure S4B). Collectively, the findings revealed that *Mettl14* mainly enhances SASP secretion in NR cells.

To determine whether the increased efficiency of iPSC generation depends on cellular senescence or the SASP, we used small molecules to treat the reprogrammed cells on day 10 with Navitoclax (also known as ABT263) to selectively reduce the viability of senescent cells by inhibiting *Bcl-2*/*Bcl-xL*/*Bcl-w* expression (Chang et al., 2016) and BAY 11-7082, an inhibitor that blocks activation of NF-κB pathway, a master regulator of the SASP (Acosta et al., 2008; Chien et al., 2011; Freund et al., 2011; Krishnan et al., 2013; Lee et al., 2012). Both inhibitors significantly reduced the number of senescent cells, as indicated by SAβG staining, which is shown in Figure 4D, and effectively blocked the upregulation of SASP gene expression by *Mettl14* or its mutant (Figure S4C). Correspondingly, these two inhibitors blocked the activation effect of *Mettl14* on reprogramming, as indicated by the number of *Oct4*-GFP^+^ and AP^+^ colonies and the percentage of *Oct4*-GFP^+^ cells (Figure 4H). These results suggested that the SASP is required for *Mettl14* to affect reprogramming.

In conclusion, the effect of *Mettl14* on reprogramming mainly depended on cellular senescence and transiently upregulated expression of SASP genes in NR cells during the late phase of reprogramming in an m^6^A-independent manner.

## Discussion

We focused on the m^6^A-independent function of *Mettl14* during *in vitro* reprogramming. *Mettl14* significantly upregulated the expression level of SASP genes during the late phase of reprogramming. It had been previously reported that in senescent cells, *Mettl14* regulated SASP genes in an m^6^A-independent manner (Liu et al., 2021). Based our METTL14 chromatin immunoprecipitation sequencing (ChIP-seq) data, we hypothesized that *Mettl14* functions as a transcription factor or co-activator, binds to promoter regions of SASP genes, and increases their expression to facilitate somatic cell reprogramming. Notably, our data supported the hypothesis that SASP genes facilitate reprogramming, which is consistent with their role during *in vivo* reprogramming (Mosteiro et al., 2016, Mosteiro et al., 2018).

The relationship between senescence and reprogramming remains controversial. OE of OSKM genes caused both cell senescence and reprogramming. It has been previously shown that long-term OE of the inflammation-related pathway *Ink4/Arf* locus, comprising *Cdkn2a-Cdkn2b* genes that encode four potent tumor suppressors, namely p16^*Ink4a*^, p19^*Arfand*^, p15^*Ink4b*^, and p21^*Cdkn1a*^, inhibited the efficiency of *in vitro* reprogramming (Dulic et al., 2000; Hong et al., 2009; Li et al., 2009). However, in the *in vivo* reprogramming system presented in the previous study, after the KD of *Ink4/Arf* pathway components, cell senescence was sharply attenuated and cell reprogramming efficiency was reduced *in vivo* (Mosteiro et al., 2016). The most widely investigated validation factor, IL-6, activates a *Jak/Stat* target, the serine/threonine kinase gene *Pim1*, resulting in a 2-fold increase in the iPSC acquisition rate (Brady et al., 2013).

The dynamic homeostatic function of senescent cells depends on their clearance by the immune system once their beneficial function has been realized (Krizhanovsky et al., 2008; Sagiv et al., 2016). Senescence induction is required for effective cell reprogramming *in vivo*, as SASP factor production promotes reprogramming of somatic cells into iPSCs in a paracrine manner (Mosteiro et al., 2016). We analyzed our RNA-seq data and found that cytokine-cytokine receptor interactions were significantly enriched with upregulated DEGs that had been induced by *Mettl14*. It has been speculated that during reprogramming, senescent cells secrete SASP factors to promote potential reprogramming of cells, enabling them to acquire pluripotency through the paracrine process.

Therefore, we believe that short-term expression of SASP genes may have beneficial effects in different systems, such as during immune surveillance and immune clearance in senescent cells, and positive effects on reprogramming efficiency during reprogramming but that their long-term expression is detrimental to the organism.

## Experimental procedures

The experimental procedures were including in supplementary information.

### Resource availability

#### Accession numbers

The sequencing datasets have been deposited in the NCBI Gene Expression Omnibus (GEO) database and are accessible through GEO: GSE196475.

## Author contributions

C.X. and L.W. designed and performed the experiments, performed the data analysis, led discussion, and wrote the manuscript; X.X. performed the bioinformatics analyses; C.X., Y.W., X.K., Y.Z., J. Sun, Y.D., Z.S., J. Shen, D.L., W.Y., Y.L., R.Z., Y.X., H.W., L.H., L.W. and S.G. contributed to the experimental work and discussion; and S.G. and L.W. supervised the study and contributed to writing. There is no conflict of interest in this article.

## Supplemental information

Document S1. Figures S1–S4, Tables S1 and S2, and supplemental experimental procedures

Document S2. Article plus supplemental information

## Footnotes

Supplemental information can be found online at https://doi.org/10.1016/j.stemcr.2022.06.012.
