# Supplementary material for: Mettl14-driven senescence-associated secretory phenotype facilitates somatic cell reprogramming
Source: Stem Cell Reports. 2022 Aug 9;17(8):1799–809. doi: 10.1016/j.stemcr.2022.06.012 (PMC9391510; doi:10.1016/j.stemcr.2022.06.012)
Supplement: Document S1. Figures S1–S4, Tables S1 and S2, and supplemental experimental procedures [file mmc1.pdf]

Stem Cell Reports, Volume *17*

Supplemental Information

Mettl14-driven senescence-associated secretory phenotype facilitates somatic cell reprogramming

Chenxiang Xi, Jiatong Sun, Xiaocui Xu, You Wu, Xiaochen Kou, Yanhong Zhao, Jiacheng Shen, Yu Dong, Kang Chen, Zhongqu Su, Dan Liu, Wen Ye, Yingdong Liu, Ran Zhang, Yiliang Xu, Hong Wang, Lujiang Hao, Li Wu, and Shaorong Gao

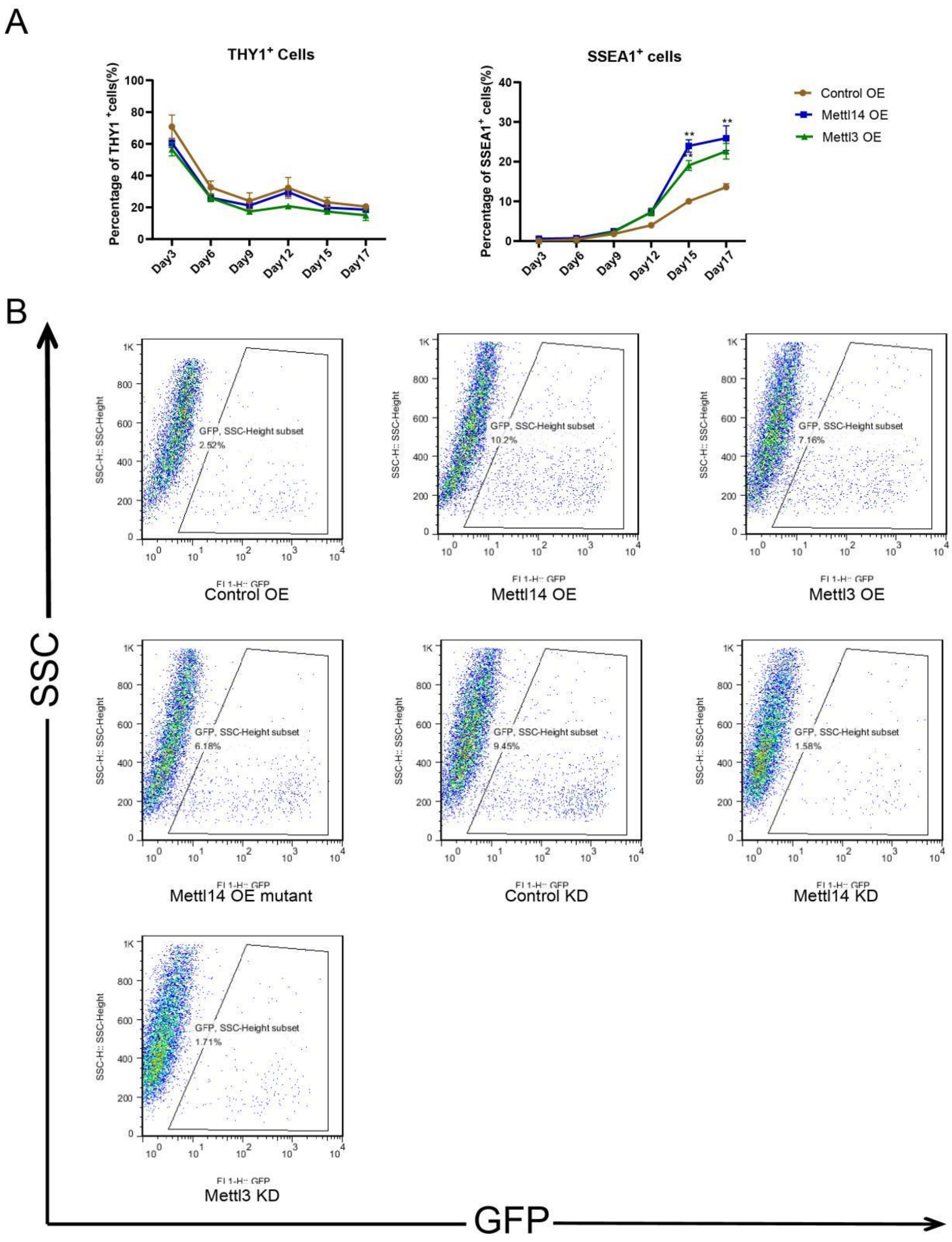

Figure S1. *Mettl14* can facilitate reprogramming in an m^{6}A-independent manner. Related to Figure 1.

(A) Kinetic changes of percentage of THY1$^+$ and SSEA1$^+$ population at indicated time points during reprogramming by FACS analysis. Data are represented as the mean ± SEM (n = 3); * $p < 0.05$, ** $p < 0.01$ by Student's *t*-test for comparison.

(B) FACS analysis showed that the proportion of *Oct4*-GFP$^+$ cells in each experimental group on Day 18 after induction.

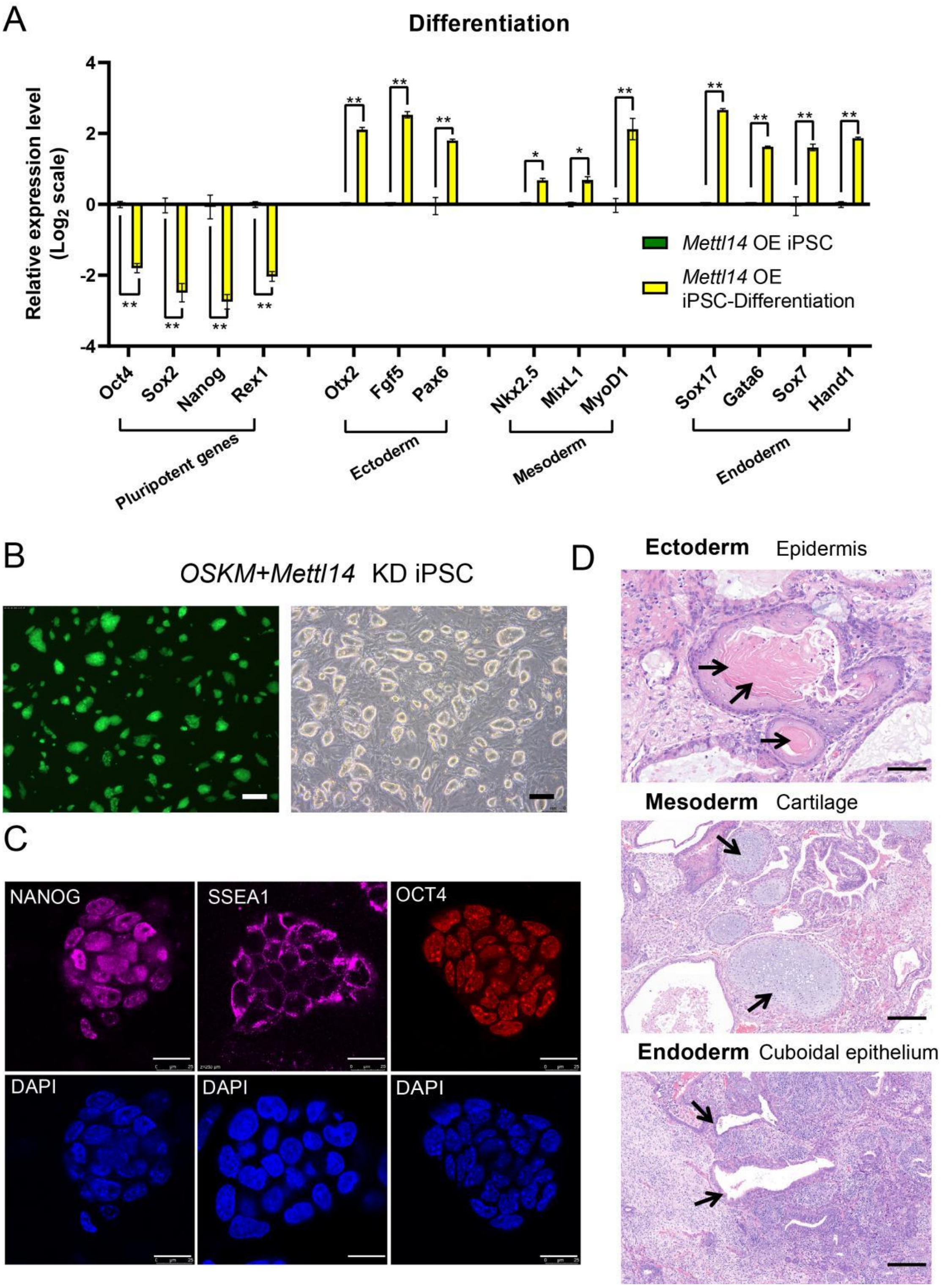

Figure S2. iPSC lines with OSKM+*Mettl14* OE or KD exhibit pluripotency. Related to Figure 2.

(A) qRT-PCR analysis shows iPSCs with OSKM+*Mettl14* OE line's embryoid bodies can subsequently differentiate and express the three embryonic marker genes. Data are represented as the mean ± SEM ($n = 3$); * $p < 0.05$, ** $p < 0.01$ by Student's *t*-test for comparison.

(B) Morphology of the iPSCs with OSKM+*Mettl14* KD lines. Scale bars, 200 µm.

(C) Immunostaining analyses for the expression of pluripotent marker genes NANOG (purple), SSEA1 (purple) and OCT4 (red), and in the iPSCs with OSKM+*Mettl14* KD lines. Nuclear staining by DAPI (blue). Scale bars, 25 µm.

(D) Haematoxylin and eosin (H&E) staining of teratomas generated by the iPSCs with OSKM+*Mettl14* KD. Scale bars, 100 µm.

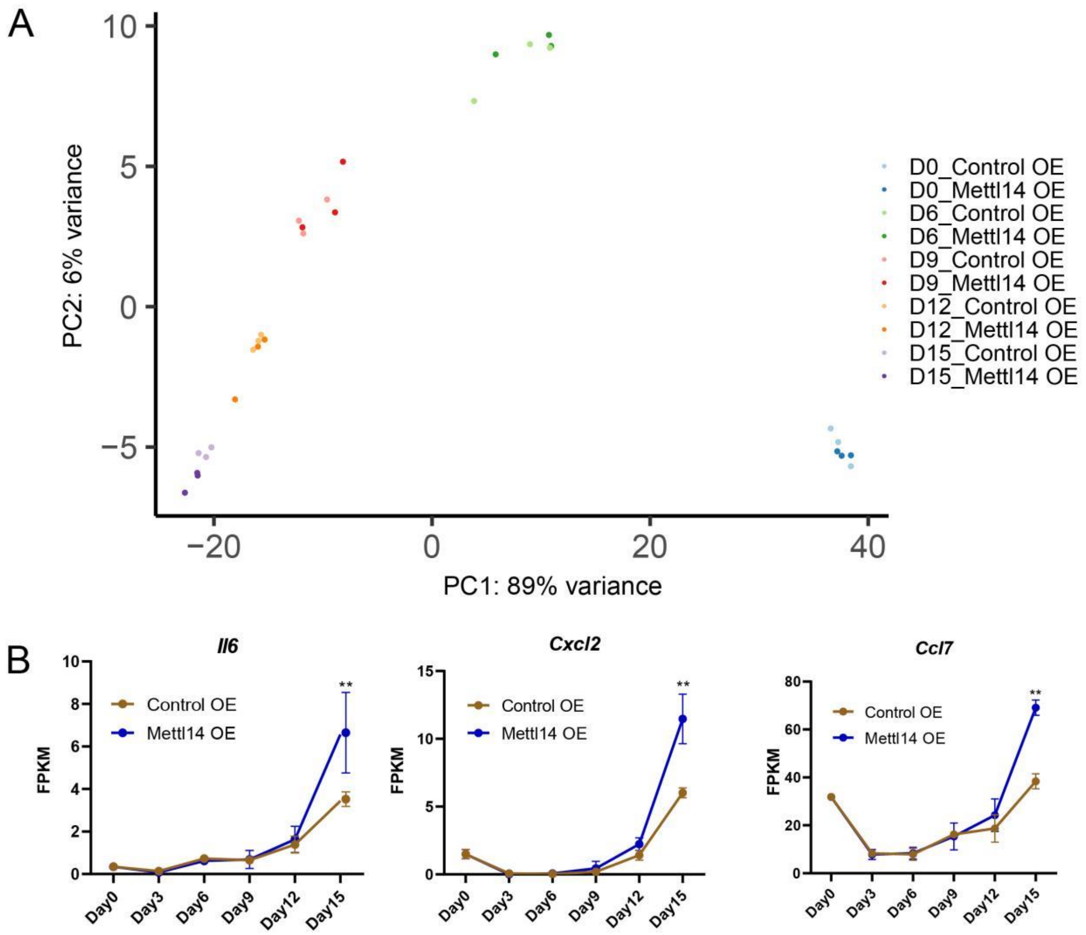

Figure S3. The expression level of SASP gene was increased after overexpression of *Mettl14*. Related to Figure 3.

(A) PCA analysis showing changes at various time points during reprogramming**.**

(B) FPKM shows changes in differential genes during reprogramming. Data are represented as the mean ± SEM ($n = 3$); * $p < 0.05$, **$p < 0.01$ by Student's *t*-test for comparison.

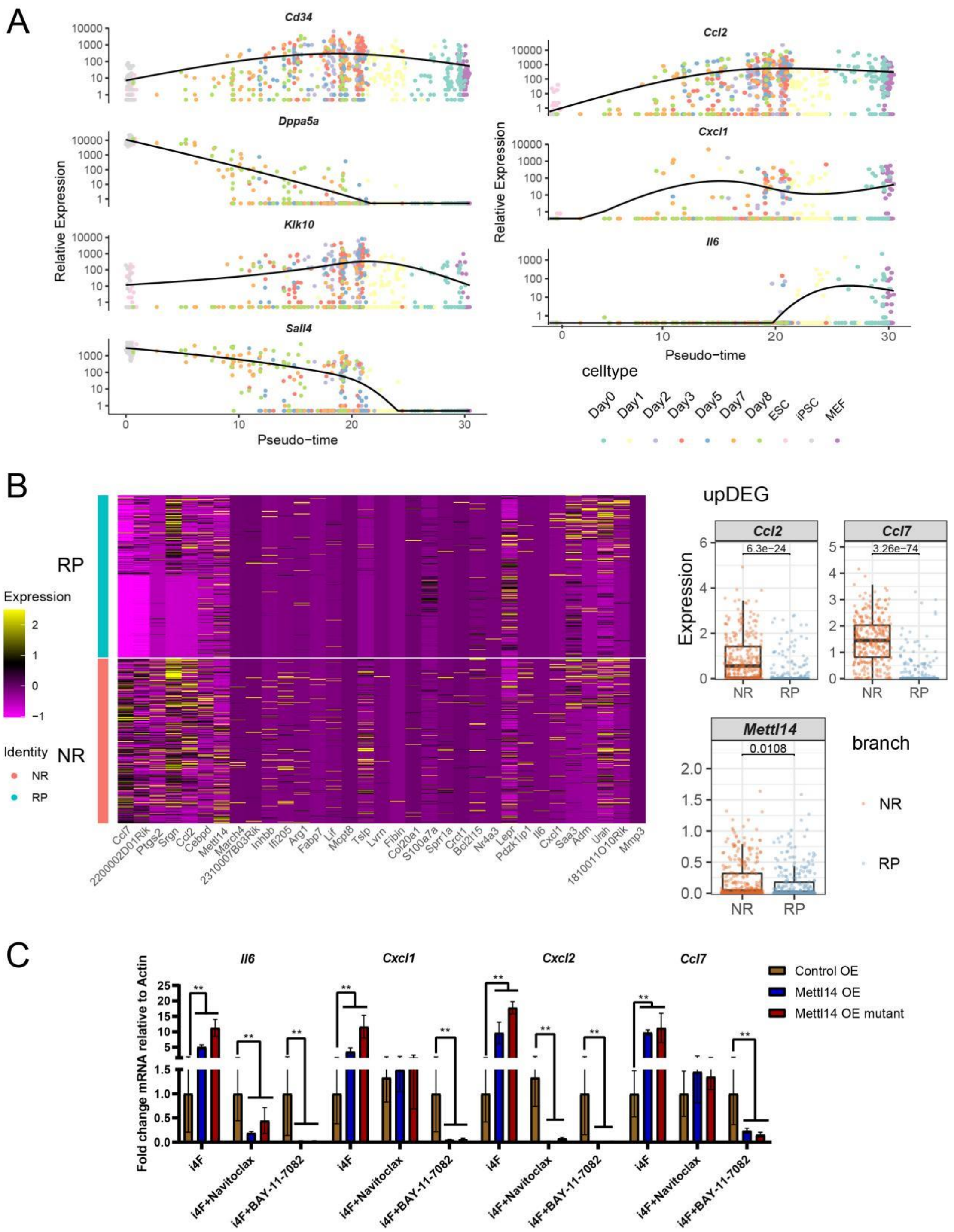

Figure S4. Reprogramming efficiency was reduced after treatment of SASP or senescence inhibitor. Related to Figure 4.

(A) Expression of the non-reprogramming (NR) branch signature genes (*Cd34* and *Klk10*) and reprogramming potential (RP) branch signature genes (*Sal4* and *Dppa5a*) along the path of the pseudotime.

(B) Identification of the differentially expressed genes between NR and RP branches using Beta-Poisson model for single-cell RNA-seq data analyses (BPSC) with adjusted p < 0.05 by Student's *t*-test for comparison..

(C) qRT-PCR analysis shows SASP gene expression in the reprogramming system with

the addition of inhibitors, shows the proportion of Fold change in these genes compared to control, represented relative to expression in *β-Actin*. Data are represented as the mean ± SEM (n = 3); * p < 0.05, **p < 0.01 by Student's *t*-test for comparison.

Table S1. Primer Sequences Used in this Paper. Related to Figure 2/S2/3/S3/4/S4.

Primer Name	Reverse Primer	Forward Primer
Oct4	CGAAGCGACAGATGGTGGTC	AGAGGATCACCTTGGGGTACA
Sox2	TGCTTTGTCCGTATCCAGTGC	AGCAATGGTTCTTATGTTGGACG
Nanog	GCAAGAATAGTTCTCGGGATGAA	CACAGTTTGCCTAGTTCTGAGG
Rex1	TCTGGGTTGTACGGGTCATAG	ATGCTACGTTCTACCGGCTTC
Otx2	GCCCTAGTAAATGTCGTCCTCTC	TATCTAAAGCAACCGCCTTACG
Fgf5	GAAGAAAACGTCGCGCTACT	GAAGCGTCTCACTCCCGAAG
Pax6	GAGTCGCCACTCTTGGCTTA	GTTGTGTGAGTAAAATTCTGGGC
Nkx2-5	CTGTCGCTTGCACTTGTAGC	GACAAAGCCGAGACGGATGG
MixL1	TCCCAGGAGTCCAACTTTGAG	ACTGAAGCTAGGTGTTTGAAGC
MyoD1	TCGAAACACGGGTCATCATAGA	CGGGACATAGACTTGACAGGC
Sox17	CCACCTCGCCTTTCACCTTTA	GATGCGGGATACGCCAGTG
Gata6	GTGGTCGCTTGTGTAGAAGGA	TTGCTCCGGTAACAGCAGTG
Sox7	CGTGTTCTGGTCACGAGAGA	ATGCTGGGAAAGTCATGGAAG
Hand1	GCATCGGGACCATAGGCAG	GGCAGCTACGCACATCATCA
Cxcl1	AACCAAGGGAGCTTCAGGGTCA	TCCAGAGCTTGAAGGTGTTGCC
Cxcl2	GGCTTCAGGGTCAAGGCAAACT	CATCCAGAGCTTGAGTGTGACG
Ccl2	GTCTGGACCCATTCCTTCTTGG	GCTACAAGAGGATCACCAGCAG
Ccl7	ATAGCCTCCTCGACCCACTTCT	CAGAAGGATCACCAGTAGTCGG
Il6	CTGCAAGTGCATCATCGTTGTTC	TACCACTTCACAAGTCGGAGGC
S100a7a	CTGGAGATGGTAGTCCTTCACC	GATAGTGTGCCTCGCTTCATGG
Calca	CTCAGATTCCCACACCGCTTAG	GCACTGGTGCAGGACTATATGC
Trp53	GAGGCCGGCTCTGAGTATACC	GTCCCAGAAGGTTCCCACTGGA
Cdkn1a	CCAATCTGCGCTTGGAGTGATAG	TCGCTGTCTTGCACTCTGGTGT
Cdkn2a	CGAATCTGCACCGTAGTTGAGC	TGTTGAGGCTAGAGAGGATCTTG
Rela	GGTCTCATAGGTCCTTTTGCGC	TCCTGTTCGAGTCTCCATGCAG
Mettl3	TGAGAGGTGGTGTAGCAACTT	CTGGGCACTTGGATTTAAGGAA
Mettl14	AGGTCCAATCCTTCCCCAGAA	GACTGGCATCACTGCGAATGA

Table S2. FPKM of genes in volcano and heat maps after overexpression of *Mettl14* on Day 15 of reprogramming in RNA-seq Data.

Gene Name	Day15 Control-1	Day15 Control-2	Day15 Control-3	Day15 Mettl14OE-1	Day15 Mettl14OE-2	Day15 Mettl14OE-3
Il6	3.10071	3.42969	3.7825	4.64235	8.43489	6.61467
Cilp	1.0128	1.50932	1.58887	3.11203	2.65677	2.40388
Saa3	8.63937	9.56221	13.9514	20.1873	29.933	11.9028
Cxcl2	6.42135	5.6963	5.94721	9.44787	12.9886	11.9734
Nr4a3	1.14686	1.78633	2.09913	4.05531	3.63952	3.6047
Ccl7	38.0989	41.6011	35.4044	72.7539	67.5895	66.9442

Lvrn	1.87122	2.45833	2.0439	3.84444	4.35767	3.27718
Srgn	73.2929	79.4373	48.5089	118.761	110.202	109.196
Ccl2	100.858	87.6044	86.9499	151.945	162.251	144.982
Col20a1	0.253553	0.424448	0.357017	0.549747	0.676647	0.550783
Fibin	2.09973	2.48539	3.49579	4.0921	4.15271	4.8317
Pdzk1ip1	11.6125	13.1095	8.74607	21.689	15.8428	16.597
S100a7a	4.316	3.54186	4.98286	8.49985	7.75266	7.92842
Ifi205	6.91898	7.39863	7.36023	10.825	12.0357	12.1769
Cxcl1	61.6357	59.0066	56.2672	97.6691	85.0323	99.7257
Lif	2.82955	2.55727	2.63662	3.25625	4.94839	4.42052
Crct1	17.9904	25.0129	15.2178	37.5422	29.7866	25.2319
F5	1.02493	1.19734	1.28256	2.24806	1.73628	1.55523
Fabp7	14.4991	12.3987	12.6412	18.2676	23.5247	19.2173
Lepr	0.941787	1.07526	1.106	1.13498	2.0702	2.02172
1810011O1	18.4608	20.5147	19.0956	31.4921	33.2224	25.2479
Inhbb	27.8833	28.4317	31.4826	47.3002	47.7918	38.4
Mcpt8	14.1991	10.7608	13.802	22.6779	18.5148	15.1304
Bcl2l15	6.09294	6.00122	5.60685	9.11008	8.56783	8.19252
Ptgs2	29.4949	30.585	35.2759	45.921	47.9811	45.6273
Tslp	6.88578	5.68467	9.0653	9.44746	11.7001	10.5428
2310007B0	3.68851	2.74692	2.72075	4.07112	4.78983	4.83833
Sprr1a	101.242	96.635	104.68	160.959	151.63	129.267
Arg1	16.4418	19.2019	17.5435	26.9708	27.3734	24.8046
Mmp3	135.827	97.636	134.692	189.83	211.784	138.147
Adm	11.7006	11.0716	14.7973	21.057	17.6963	15.5685
Ces2f	11.1061	12.7747	9.26462	16.4765	17.8925	13.4787
Urah	21.6548	22.2704	24.2253	31.4051	33.0631	33.3812
2200002D0	88.0926	89.1785	70.3239	126.75	106.258	132.082
Cebpd	20.8436	22.8618	24.0382	33.7725	32.3735	31.3504

SUPPLEMENTAL EXPERIMENTAL PROCEDURES

Mice

Oct4-GFP^{+} (OG2) mice (Jackson laboratory code 004654) were crossed with R26rtTA; Col1a1-4F2A mice (Jackson laboratory code 011004) to obtain Rosa26-M2rtTA; *Col1a1*-4F2A; *Oct4*-GFP mice. The pathogen-free mice were housed in SPF-level mouse houses at Tongji University. All of our animal research methods were in accordance with Tongji University guidelines for the use of laboratory animals.

Cell culture for MEFs, feeders and iPSCs

Mouse embryonic fibroblasts (MEFs) for iPSC induction were derived from 12.5-13.5 days embryos (Embryos were derived from the above transgenic mice), and MEFs were cultured in Dulbecco's Modified Eagle Medium (DMEM) (Gibco C11960500BT) supplemented with 1 mM L-glutamine (EmbryoMax TMS-002-C), and 10% (vol/vol) fetal bovine serum (FBS) (Gibco 10270-106). Feeder was obtained by treating the obtained MEFs with mitomycin C for three hours to render them incompetent for proliferation. iPSCs was cultured on feeder in embryonic stem cell medium (ESM), a medium of DMEM containing 1% nonessential amino acid (NEAA), stock (Merck Millipore TMS-001-C), 1 mM L-glutamine (Merck Millipore TMS-002-C), 15% (v/v)

FBS (Gibco 16000-44), 0.1 mM *β*-mercaptoethanol (Merck Millipore ES-007-E) and 1000 U/ml leukaemia inhibitory factor (LIF) (Merck Millipore ESGRO 1107).

iPSCs derivation

The HEK293T cells were transfect with overexpression or knockout plasmids, lentivirus packaging plasmid psPAX2 and pMD2G using VigoFect transfection reagent (Vigorous Biotechnology), and replaced fresh ESM 10 hours after transfection. After 48 hours of transfection, the virus-containing supernatant was collected and filtered using a 0.45 µm filter (Millex SLHV033RB). For overexpression, the starting MEFs were seeded in 12-well plates at a density of 0.6-0.8×10^4 cells/well, and for knockdown, the starting MEFs at a density of 1.2-1.3×10^4 cells/well. Cells were then infected with collected virus-containing cultures for 8-12 hours. Infected MEFs were cultured in ESM supplemented with 1 µg/mL Dox for 16-18 days. After clonal morphology formation, culture was continued in ESM without Dox for 2-3 days and individual clonal colonies were picked to establish iPS cell lines.

The iPS colonies will appear after two weeks of Dox treatment, and counted under the microscope at the indicated time points. The number of *Oct4*-GFP$^+$ colonies was shown as the number in each well in a 12-well plate.

Flow cytometry analysis

Induced cells at indicated time points were washed once with DPBS, digested with trypsin and EDTA (TE) solution and neutralized with serum-containing medium. The cells were centrifuged and resuspended with FACS buffer (PBS+0.1% BSA) and filtered for FACS assay. If antibody incubation is required, the cells were resuspended at appropriate density with FACS buffer. The antibodies were added according to the antibody instruction, and incubate for 30 minutes on ice protecting from light. The cells then washed using FACS buffer once and filtered. Flow analysis instrument used in this experiment was provided by FACS Calibur (BD, CA 95131), and the sorting flow analyzer was provided by FACS Aria II (BD).

Cell growth curve

MEFs were seeded in 12-well plates and changed fresh ESM with Dox every other day. The cells were harvested every three days until the end of reprogramming induction by withdrawal of Dox. Three replicates were containing for each sample at each time point. The cells were counted by hemocytometer.

RNA isolate and real time PCR

The cell samples were lysed with RNAiso Plus. Total RNA was extracted with TRNzol Universal Reagent (Tiangen), reverse transcribed to cDNA using 5× All-In-One TE MasterMix (Abm Cat. G490), and reverse transcribed using Eppendorf AG (223331). qRT-PCR was performed in three replicates per sample, using the ΔCT or ΔΔCT method to normalize the data, and the internal reference was *β-actin*.

Alkaline phosphatase (AP) staining

At the end of reprogramming, the cells in the 12-well plate were washed once with DPBS, then fixed in 10% formaldehyde for 5 minutes at room temperature, and washed three times

with DPBS. The fixed cells were stained using the Beyotime BCIP/NBT alkaline Phosphatase Color Development Kit (Cat. No. C3206). The staining solution is incubated for 30 minutes at room temperature and protected from light, and the staining is stopped by washing twice with DPBS.

Senescence *β*-Galactosidase (*β*-Gal) Staining

The cells in the cell culture plate were washed once with DPBS. The Senescence *β*-Galactosidase Staining Kit (Beyotime Cat.No.C0602) was used for staining. Briefly, the cells were fixed by *β*-galactosidase staining fixative at room temperature for 15 minutes. Then the cell fixative was aspirated and was washed with PBS 3 times and stained with staining working solution at 37 °C overnight. The stain was removed at the end of staining, and the cells were washed once with DPBS to abort the staining, and store at 4 °C. Taking photos of stained cells using Microsystems CMS GmbH (Leica, D-35578 Wetzlar)

Immunofluorescence (IF) staining

The iPSCs was planted in a 12-well plate with slides, and the colonies were obtained at an appropriate size. The cells were fixed using 4% paraformaldehyde (PFA) for 1 hour at room temperature, rinsed in PBS and permeabilized with 0.3% Trition X-100 in DPBS for 15 minutes at room temperature. The cells were washed with DPBS and blocked with blocking solution (2.5% BSA in DPBS) at room temperature for 1 hour. The samples were incubated with primary antibody OCT4 (1:1000, Santa Cruz, SC-5279), NANOG (1:1000, Cosmo Bio, RCAB001P) diluted in blocking solution incubate for 2 hours at room temperature, then washed three times with DPBS, and incubated secondary antibody Alexa Fluor 594 donkey anti-mouse IgG (Thermo Fisher, A21203), or Alexa Fluor 594 donkey anti-rabbit IgG (Thermo Fisher, A21207). DAPI (1 µg/mL, Merck Millipore) diluted in DPBS was used to labeled DNA for 5-10 min at room temperature. The samples were imaged using Leica Microsystems (Type: TCS SP8) confocal microscope.

Embryoid body (EB) differentiation

The iPSCs were trypsinized and plated onto tissue culture plates for 30 minutes to deplete feeder cells. The supernatant cells were collected and incubated for two days with 5×10^4 cell suspensions per drop and transferred to ultra-low cluster plates (Costar). The cells were cultured in ESM without LIF. EBs was collected after 5-10 days and seeded in gelatin-coated tissue culture dishes for 14 days. Total RNA of the cells was then extracted and the analyzed of marker genes in the three germ layers was detected by qRT-PCR.

Teratoma formation

The iPSCs were trypsinized and resuspended with DPBS at $2\text{-}3 \times 10^6$ and injected subcutaneously in the groin of SCID mice. Three to four weeks later, tumor-like growths were seen at the injection site. Mice were dislocated and executed at the neck. The teratomas were stripped, placed in 10% formaldehyde solution, fixed overnight at room temperature, stained for hematoxylin-eosin staining, and the sections were observed under a microscope and identify the iconic tissue structures of the skin epithelium (ectoderm), cartilage (mesoderm) and cuboidal epithelium (endoderm), and take photos to record.

RNA-sequencing and data processing

Collect RNA samples during reprogramming different time points, three biological replicates for each sample. Total RNA was isolated using QIAGEN RNasy Kit (14104, Germantown, US). The RNA sequencing libraries were generated using a KAPA Stranded RNA-Seq Kit Illumina platform (KK8440, Wilmington, US). Paired- end 150-bp sequencing was further performed on a HiSeq 2500 (Illumina) at Berry Genomics Corporation.

RNA-seq data were first subjected to Trim_galore (version 0.6.4) for adaptor trimming as well as quality control with the parameters --paired -j 7 --basename. The trimmed paired-end reads were then aligned to mm9 reference genome with random chromosome cleaned by STAR (version 2.7.3a) under the parameters --runThreadN 30 --runMode alignReads --outSAMtype BAM SortedByCoordinate --outSAMstrandField intronMotif. The expression of genes was quantified as FPKM by Cufflinks (version 2.2.1). For the downstream data analyses, FPKM values were averaged for each gene between replicates. The RefSeq gene annotation files were downloaded from UCSC. For genes with multiple isoforms, the longest transcripts were selected. The R package DESeq2 (version 1.26.0) were used for gene differential expression analysis. Fold change > 1.5 and FDR < 0.05 were used as cutoff for down-regulated and up-regulated genes. To perform PCA analysis, read counts per gene were summarized by the featureCounts function from Subread package. Then the raw counts were subjected to Variance Stabilizing Transformation by vst function from DESeq2 package, and then PCA values were calculated by plotPCA function from DESeq2 package.

Gene Ontology (GO) enrichment was analyzed by Gene Ontology biological process (GO_BP) and Kyoto Encyclopedia of Genes and Genomes (KEGG) in the Database for Visualization, Annotation and Integrated Discovery (DAVID) web- accessible tool. Gene ontology terms for each function cluster were summarized to a representative term, and p-values were plotted to show the significance.

ELISA

The kit used for the experiment was ABclonal ELISA Kits (Cat.No:RK00038), Specific experimental procedures refer to the instructions.

IL6 cytokines

Add 0.05ng/ml Recombinant Murine IL6 (PEPROTECH, Catalog #216-16) to the cells at different periods of the reprogramming process to explore the effect of IL6 on the reprogramming process, and count the reprogramming efficiency of each experimental group after completing the induction.

Conditional medium induce

The conditional medium of each experimental group during reprogramming was collected. The collected medium was added to the untransfected reprogrammable MEF cells (The induction time of transfected reprogrammable MEF cells(conditional medium-providing group) was two days earlier than that of non-transfected reprogrammable MEF cells, and 1 μg/mL Dox supplementation to non-transfected MEF cells was required after the addition of cell conditional medium), and the cells were cultured until the conditional medium-providing group

completed the reprogramming induction process, and the untransfected reprogrammable MEF cells were assayed for reprogramming efficiency.

Western blot analysis

Cells were washed once with DPBS, digested with trypsin and EDTA (TE) solution, lysed with a cell lysis solution containing 20 mM Tris (pH 7.5), 150 mM NaCl, 1% Triton X-100, and various protein inhibitors such as sodium pyrophosphate, *β*-glycerophosphate, EDTA, leupeptin solution. Cells were lysed by adding 1 mL of lysis buffer per 10^7 cells on ice for half an hour and then ultrasonicated. Protein concentration was determined with Enhances BCA Protein Assay Kit (Beyotime, Cat No.P0010). The samples were boiled to 100 °C for 10-15 minutes in loading buffer (EpiZyme, LT101S) with 2% *β*-mercaptoethanol (Amersham, CT). Western blot experiment (Protein electrophoresis, protein transfer, antibody containment) was performed according to Abcam Western blot protocol. Primary antibody use: anti-METTL14 (Sigma, Cat. No. HPA038002; 2 µg for immunoprecipitation, 1:1,000 for western blots), anti-NF-κB P65 (Cell Signaling, Cat. No. 8242; 1:1,000 for western blots), anti-P16 (Santa Cruz Biotechnology, Cat. No. sc-56330; 1:1,000 for western blots), anti-P21 (Abcam, Cat. No. 7960; 1:1,000 for western blots), anti-*β-Actin* (Merck, Cat. No. A1978, 1:1,000 for western blots). The secondary antibody used for Western blot were ECL peroxidase-labelled sheep anti-mouse antibody (GE Healthcare, NA931V) or HRP-labelled goat anti-rabbit antibody (Beyotime, A0208).

Single cell RNA-seq data analysis

Raw read counts of single cell RNA-seq data for samples of iPSC generation process were downloaded from GSE103221 (Guo et al., 2019). The raw read counts were then converted to HDF5 format by the count-to-h5 function from MAESTRO package (version 1.3.1). The expression matrices were then loaded into R (version 4.0.5) with Seurat (version 4.0.3). The Seurat object was then subjected to pseudotime analysis by Monocle (v.2.18.0). The gene pseudotime plots were generated by plot_genes_in_pseudotime function from Monocle package, and the heatmaps were generated by DoHeatmap function from Seurat package.

Statistical Analysis

Results were represented as the mean ± SEM of independent experiments. Significance was determined with Student's *t* tests.

Supplemental reference

Guo, L., Lin, L., Wang, X., Gao, M., Cao, S., Mai, Y., Wu, F., Kuang, J., Liu, H., Yang, J., *et al.* (2019). Resolving Cell Fate Decisions during Somatic Cell Reprogramming by Single-Cell RNA-Seq. Mol Cell *73*, 815-829 e817.
