# Supplementary material for: Mettl14-driven senescence-associated secretory phenotype facilitates somatic cell reprogramming
Source: Stem Cell Reports. 2022 Aug 9;17(8):1799–809. doi: 10.1016/j.stemcr.2022.06.012 (PMC9391510; doi:10.1016/j.stemcr.2022.06.012)
Supplement: Document S2. Article plus supplemental information [file mmc2.pdf]

Stem Cell Reports
Report
OPEN ACCESS

Mettl14-driven senescence-associated secretory phenotype facilitates somatic cell reprogramming

Chenxiang Xi,[1,5] Jiatong Sun,[2,5] Xiaocui Xu,[2] You Wu,[2] Xiaochen Kou,[2,3] Yanhong Zhao,[2,3] Jiacheng Shen,[2] Yu Dong,[2] Kang Chen,[2] Zhongqu Su,[2] Dan Liu,[2] Wen Ye,[2] Yingdong Liu,[2] Ran Zhang,[4] Yiliang Xu,[2] Hong Wang,[2,3] Lujiang Hao,[1,*] Li Wu,[2,*] and Shaorong Gao[2,3,*]
[1]School of Bioengineering, Qilu University of Technology (Shandong Academy of Sciences), Jinan 250353, China
[2]Shanghai Key Laboratory of Maternal Fetal Medicine, Clinical and Translational Research Center of Shanghai First Maternity and Infant Hospital, School of Life Sciences and Technology, Tongji University, Shanghai 200092, China
[3]Frontier Science Center for Stem Cell Research, School of Life Sciences and Technology, Tongji University, Shanghai 200092, China
[4]Anhui Toneker Biotechnology Co., Ltd., Jinzhai, Anhui 201615, China
[5]These authors contributed equally
*Correspondence: lujiang_hao@qlu.edu.cn (L.H.), 14_wuli@tongji.edu.cn (L.W.), gaoshaorong@tongji.edu.cn (S.G.)
https://doi.org/10.1016/j.stemcr.2022.06.012

SUMMARY

The METTL3-METTL14 complex, the "writer" of N^6-methyladenosine (m^{6}A), plays an important role in many biological processes. Previous studies have shown that *Mettl3* overexpression can increase the level of m^{6}A and promote somatic cell reprogramming. Here, we demonstrate that *Mettl14*, another component of the methyltransferase complex, can significantly enhance the generation of induced pluripotent stem cells (iPSCs) in an m^{6}A-independent manner. In cooperation with *Oct4*, *Sox2*, *Klf4*, and *c-Myc*, overexpressed *Mettl14* transiently promoted senescence-associated secretory phenotype (SASP) gene expression in non-reprogrammed cells in the late stage of reprogramming. Subsequently, we demonstrated that interleukin-6 (IL-6), a component of the SASP, significantly enhanced somatic cell reprogramming. In contrast, blocking the SASP using a senolytic agent or a nuclear factor κB (NF-κB) inhibitor impaired the effect of *Mettl14* on reprogramming. Our results highlight the m^{6}A-independent function of *Mettl14* in reprogramming and provide new insight into the interplay between senescence and reprogramming *in vitro*.

INTRODUCTION

The N^6-methyladenosine (m^{6}A) modification is linked to human diseases because it affects multiple biological processes, including the cell cycle, fate determination, and homeostasis (Batista et al., 2014; Geula et al., 2015; Wang et al., 2014; Wen et al., 2018). Three different classes of protein factors are involved in the function of m^{6}A modification: writers (adenosine MTases), erasers (m^{6}A-demethylating enzymes), and readers (m^{6}A-binding proteins) (Zhao et al., 2020). Deposition of m^{6}A is catalyzed by the METTL3-METTL14 methyltransferase complex (MTC), and removal of m^{6}A mainly depends on alpha-ketoglutarate-dependent dioxygenase AlkB homolog 5 (ALKBH5) and fat mass and obesity-associated protein (FTO) (Zaccara et al., 2019). In the m^{6}A MTC complex, METTL3 mainly serves as the catalytic core, while METTL14 serves as the RNA-binding platform (Wang et al., 2016).

Reprogramming of somatic cells into induced pluripotent stem cells (iPSCs) by Yamanaka factors (*Oct4*, *Sox2*, *Klf4*, and *c-Myc*, known as OSKM) provides a system to study the molecular mechanisms of the cell-fate transition (Kang and Gao, 2012; Takahashi and Yamanaka, 2006). The role of m^{6}A modifications in the generation of iPSCs is controversial, which may be due to the intricate biological functions of m^{6}A (Aguilo et al., 2015; Chen et al., 2015). Increased m^{6}A deposition by modulated METTL3 promotes cell reprogramming into pluripotent cells (Chen et al., 2015), but in conjunction with ZFP217 expression, downregulated METTL3 expression also contributes to reprogramming (Aguilo et al., 2015). It remains unclear how m^{6}A modulates reprogramming and whether other factors of MTC affect reprogramming or whether other mechanisms are involved.

During reprogramming, accumulated damaged DNA and abnormal DNA replication cause cellular senescence. A notable signature of senescent cells is increased expression of cell-cycle-inhibitory proteins, such as p16^{Ink4a} and p21^{Cdkn1a} (Alcorta et al., 1996). In addition, senescent cells exhibit noncellular autonomous activities, such as secretion of inflammatory cytokines and chemokines (Acosta et al., 2013), which are together defined as the senescence-associated secretory phenotype (SASP) (Lopes-Paciencia et al., 2019). Transient expression of the SASP facilitates proper tissue development, tissue repair, and immune cell recruitment, but its persistent expression may induce chronic inflammation and lead to diseases associated with aging (Fitzner et al., 2012; Krizhanovsky et al., 2008; Yun et al., 2015). In senescent cells, SASP-mediated immune clearance depends on METTL14 in an m^{6}A-independent manner (Liu et al., 2021).

The effect of senescence on reprogramming is still unclear. In an *in vivo* reprogramming system, induced Yamanaka factors drive cellular senescence and SASP production,

This is an open access article under the CC BY license (http://creativecommons.org/licenses/by/4.0/).

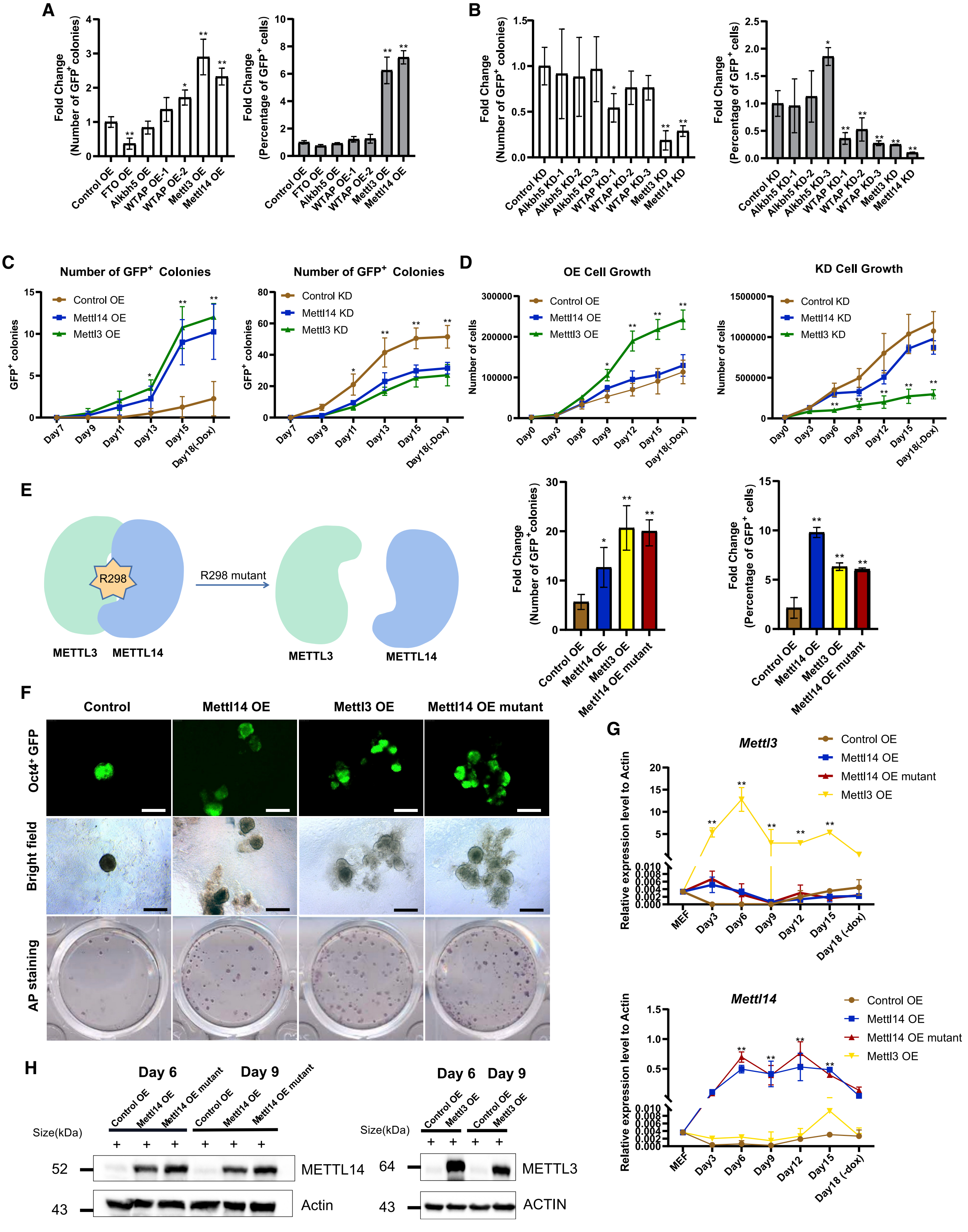

(legend on next page)

which can effectively promote reprogramming (Mosteiro et al., 2016, 2018). In addition, the most prominent cytokine in the SASP, interleukin-6 (IL-6), enhances iPSC generation, serving as an extrinsic replacement for stably transduced transcription factors such as the potent oncogene *c-Myc* (Brady et al., 2013). In this study, we identified *Mettl14* as a strong activator of *in vitro* reprogramming via transient upregulation of SASP genes in an m^6A-independent manner.

RESULTS

Mettl14 can facilitate reprogramming in an m^6A-independent manner

To investigate the effect of m^6A level on the reprogramming process, we screened the m^6A writers and erasers using reprogrammable mouse embryonic fibroblasts (MEFs) from *Rosa26*-M2rtTA; *Col1a1*-4F2A; *Oct4*-GFP$^+$ transgenic mice (Carey et al., 2010). Exogenous doxycycline (Dox) can induce the expression of OSKM and reprogram MEFs into *Oct4*-GFP$^+$ iPSCs, as previously reported (Wu et al., 2017, 2021). We found that *Mettl3* and *Mettl14* expression significantly increased the number of *Oct4*-GFP$^+$ colonies and the percentage of *Oct4*-GFP$^+$ cells (Figures 1A and S1B), while knockdown (KD) of MTC component expression remarkably reduced the number of colonies and cells (Figures 1B and S1B). Compared with the control group (overexpressing an empty vector), *Mettl14* or *Mettl3* overexpression (OE) accelerated *Oct4*-GFP$^+$ colony formation and led to an approximately 6-fold increase in the number of *Oct4*-GFP$^+$ colonies (Figure 1C, left panel), but the increase in *Oct4*-GFP$^+$ colonies was delayed by *Mettl14* or *Mettl3* expression KD, and ultimately, the number of *Oct4*-GFP$^+$ colonies was reduced (Figure 1C, right panel). To further investigate the impact of *Mettl14* and *Mettl3* on the reprogramming process, we monitored intermediate population progression. Neither *Mettl14* nor *Mettl3* affected the THY1$^+$ population transition to the THY1$^-$ population (Figure S1A, left panel), while the SSEA1$^+$ population was significantly increased by *Mettl14* or *Mettl3* (Figure S1A, right panel). These findings suggested that *Mettl14* and *Mettl3* are involved in cell acquisition of pluripotency during reprogramming.

Although the ability to promote reprogramming was comparable, the effects of *Mettl14* or *Mettl3* on the proliferation of reprogramming cells were very different. *Mettl3* significantly accelerated cell proliferation, but *Mettl14* negligibly affected cell proliferation, during reprogramming (Figure 1D).

To further examine whether the effects of *Mettl14* are dependent on the m^6A modification, we induced the expression of the *Mettl14* R298E mutant, which did not bind adequately with METTL3 and resulted in disruption of MTC activity (Figure 1E) (Wang et al., 2016). The *Mettl14* R298E mutant also led an increase in the number of *Oct4*-GFP$^+$ colonies and percentage of *Oct4*-GFP$^+$ cells (Figure 1E, middle and right panels), as well as alkaline phosphatase-positive (AP$^+$) colonies (Figure 1F, bottom panel).The OE levels of *Mettl3* and *Mettl14* during reprogramming were detected at the RNA (Figure 1G) and protein levels (Figure 1H),

Figure 1. *Mettl14* can facilitate reprogramming in an m^6A-independent manner

(A) The number of *Oct4*-GFP$^+$ colonies was counted, and the percentage of *Oct4*-GFP$^+$ cells in the overexpression (OE) group was analyzed by FACS 18 days after induction (starting MEF density was 8,000 cells/well in a 12-well plate). The data are presented as average fold change of *Oct4*-GFP$^+$ colonies (left panel) or percentage of *Oct4*-GFP$^+$ cells (right panel) ± SEM (n = 3); *p < 0.05, **p < 0.01 by Student's t test performed for comparison (control OE, empty vector control).

(B) The number of *Oct4*-GFP$^+$ colonies was counted, and the percentage of *Oct4*-GFP$^+$ cells in the knockdown (KD) group was analyzed by FACS 18 days after induction (the MEF starting density was 12,000 cells/well of a 12-well plate). The data are presented as average fold change of *Oct4*-GFP$^+$ colonies (left panel) or percentage of *Oct4*-GFP$^+$ cells (right panel) ± SEM (n = 3); *p < 0.05, **p < 0.01 by Student's t test performed for comparison (control KD, scramble short hairpin RNA [shRNA] control).

(C) The number of *Oct4*-GFP$^+$ colonies formed was facilitated by *Mettl14* or *Metttl3* OE. The opposite effect was observed after the expression of each was knocked down. The MEF starting density was 8,000 cells/well for OE and 12,000 cells/well for KD in a 12-well plate. The data are presented as the means ± SEM (n = 3); *p < 0.05, **p < 0.01 by Student's t test performed for comparison.

(D) Cells were counted at different time points during reprogramming, and growth curves were plotted. The data are presented as the means ± SEM (n = 3); *p < 0.05, **p < 0.01 by Student's t test performed for comparison.

(E) Schematic representation of the mutation at the *Mettl14* R298E locus (left panel). Estimated reprogramming efficiency of R298E mutant-expression cells as determined by the number of *Oct4*-GFP$^+$ colonies formed and the percentage of *Oct4*-GFP$^+$ cells (middle and right panels) (the MEF starting density was 6,000 cells/well in a 12-well plate). The data are presented as the means ± SEM (n = 3); *p < 0.05, **p < 0.01 by Student's t test performed for comparison.

(F) Morphology of the *Oct4*-GFP$^+$ primary colonies (top and middle panels). Representative image of AP-stained plates captured 18 days after induction (bottom panel). Scale bars, 400 μm.

(G) qRT-PCR analysis showing the expression level of *Mettl3* and *Mettl14* in the iPSCs at RNA levels. The data are presented as the means ± SEM (n = 3); *p < 0.05, **p < 0.01 by Student's t test performed for comparison.

(H) Western blot showing the expression level of *Mettl3* and *Mettl14* in the iPSCs at protein levels. ACTIN is used as loading control.

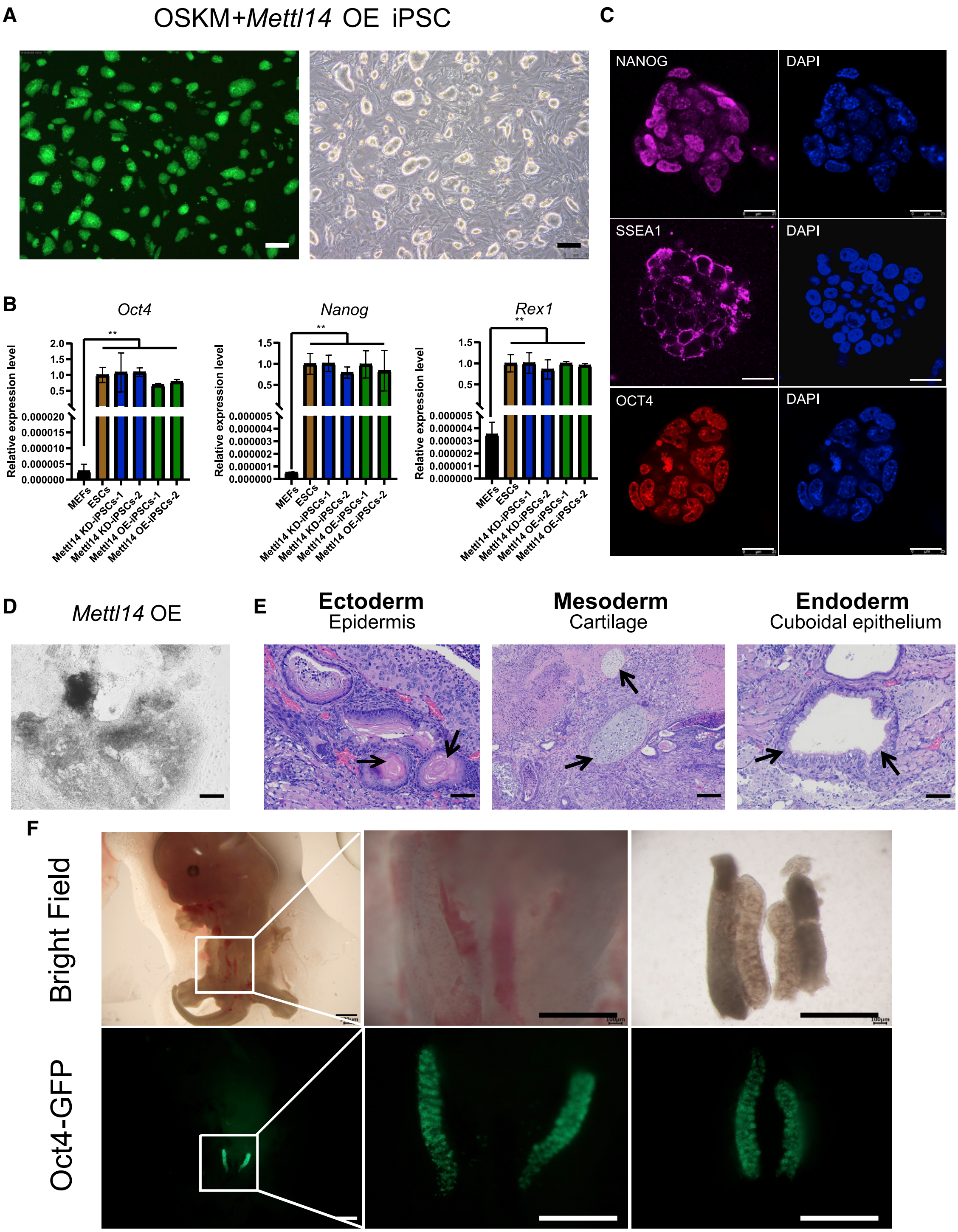

Figure 2. iPSC lines with OSKM+*Mettl14* OE exhibit pluripotency

(A) Morphology of the iPSCs with OSKM+*Mettl14* OE lines. Scale bars, 200 μm.

(B) qRT-PCR analysis showing pluripotent gene expression in the iPSCs with OSKM+*Mettl14* OE/KD relative to their expression in MEFs and ESCs. The data are presented as the means ± SEM (n = 3); *p < 0.05, **p < 0.01 by Student's t test performed for comparison.

(C) Immunostaining analyses for the expression of pluripotent marker genes NANOG (purple), SSEA1 (purple), and OCT4 (red) and in the iPSCs with OSKM+*Mettl14* OE lines. Nuclear staining by DAPI (blue). Scale bars, 25 μm.

(D) Differentiation of the embryoid bodies of the iPSCs with OSKM+*Mettl14* OE line showing the differentiation potential. Scale bars, 200 μm.

(legend continued on next page)

respectively, and the expression levels of *Mettl3* and *Mettl14* were significantly increased compared with the control group.

iPSC lines with OSKM + *Mettl14* OE exhibit pluripotency

Established iPSC lines derived upon OE of *Mettl14* (OSKM+ *Mettl14* OE iPSCs) exhibited typical embryonic stem cell (ESC) morphology with large nuclei and nucleoli, a compact appearance, and clear boundaries (Figure 2A). Quantitative reverse transcription PCR (qRT-PCR) showed that iPSCs with OSKM+*Mettl14* OE were comparable to ESCs in terms of mRNA expression levels of pluripotency genes such as *Oct4*, *Nanog*, and *Rex1* (Figure 2B), and protein expression levels of pluripotent genes, as shown by immunofluorescence staining (Figure 2C) (Kang et al., 2009).

To further demonstrate the quality of the iPSCs with OSKM+*Mettl14* OE, we performed *in vitro* and *in vivo* differentiation assays to detect their differentiation potential (Kang et al., 2009). Through embryoid body (EB)-mediated *in vitro* differentiation, the markers of the three germ layers in differentiated cells were significantly upregulated (Figures 2D and S2A). After subcutaneous injection of iPSCs with OSKM+*Mettl14* OE in nude mice, teratomas formed within the three germ layer tissues, which consisted of skin epithelium (ectoderm), cartilage (mesoderm), and cuboidal epithelium (endoderm) (Figure 2E) (Le et al., 2014). Furthermore, the iPSC lines with OSKM+*Mettl14* OE were successfully integrated into the gonads of chimeric mice, as shown by chimera formation assay (Figure 2F).

The iPSC lines derived from *Mettl14*-KD cells (OSKM+ *Mettl14*-KD iPSCs) also exhibited an ESC-like morphology (Figure S2B), expressed pluripotent genes (Figure S2C), and differentiated into three germ layers in the teratoma assay (Figure S2D).

Increased expression level of SASP genes after *Mettl14* OE

To investigate how *Mettl14* facilitates reprogramming, we collected samples with or without *Mettl14* OE at various time points during reprogramming and performed RNA sequencing (RNA-seq). We performed a principal-component analysis (PCA) to compare the transcriptomes of the reprogramming cells at the indicated time points. The PC1 axis was dominated by differences among reprogramming intermediate cells. Specifically, the cells showed clear stepwise transcriptome changes during the reprogramming of MEFs (Figure S3A). Volcano plots showed that exogenous *Mettl14* treatment resulted in the upregulation of 37 differentially expressed genes (DEGs) (fold change [FC] > 1.5, false discovery rate [FDR] < 0.05) and downregulation of 33 DEGs on day 15, compared with the control group (Figure 3A).

Gene Ontology (GO) enrichment analysis showed that the DEGs increased by *Mettl14* OE were mainly enriched in immune response and cytokine-cytokine receptor interactions (Figure 3B). To understand these data, we searched the literature about immunity in cell reprogramming and *Mettl14*-related phenotypes. It has been reported that, in senescent cells, m^6A-independent genome-wide *Mettl3* and *Mettl14* redistribution drives the SASP (Liu et al., 2021). Therefore, we analyzed the reported SASP genes (Andriani et al., 2016; Marcheggiani et al., 2021; Mosteiro et al., 2016; Suvakov et al., 2019; You et al., 2019) in our data and found that a number of SASP genes was upregulated upon treatment with exogenous *Mettl14*, as shown in the related heatmap (Figure 3C). Surprisingly, we also found that the upregulated DEGs were significantly enriched for SASP genes (7 of 37 upregulated DEGs are SASP genes, Fisher's exact p value < 2.573e-10; Figure 3A).

To investigate how SASP genes are regulated during reprogramming, we plotted their dynamic expression levels. The expression levels of SASP genes, such as *Il6*, C-X-C motif chemokine ligand 2 (*Cxcl2*), and C-C motif chemokine ligand 7 (*Ccl7*), were increased after day 12 and peaked on day 15 (Figure S3B). To confirm the effects of *Mettl14* on late-phase reprogramming, we performed qRT-PCR assays to ascertain the expression levels of SASP genes in cells' expression of wild-type or mutant *Mettl14* from days 3 to 18 and in iPSCs. As shown in Figure 3D, the expression levels of SASP genes peaked on day 15 and then dramatically decreased on day 18. These SASP genes were negligibly expressed or not expressed even in the established iPSC line cells (Figure 3D).

To confirm that SASP factors were secreted, we performed ELISAs to examine the secreted protein levels of IL-6, CXCL2, and CXCL1 in the late reprogramming period. The level of these factors in the medium of cells expressing either *Mettl14* wild-type or the mutant were significantly higher than those in the control group on day 15 (Figure 3E). In general, *Mettl14* transiently upregulated the expression levels of SASP genes in the late phase of reprogramming in an m^6A-independent manner.

SASP genes are key factors in regulating reprogramming efficiency

Considering these findings, we hypothesized that SASP factors were secreted from intermediate cells during the phase of reprogramming. We collected the conditioned medium of the late reprogrammed cells and used it to

(E) Hematoxylin and eosin (H&E) staining of teratomas generated by the iPSCs with OSKM+*Mettl14* OE. Scale bars, 100 μm.
(F) Representative photos showing the contribution and spatial distribution of *Oct4*-GFP^+ cells in the gonads of the iPSCs with OSKM+ *Mettl14* OE-derived chimeric embryos on embryonic day 12.5 (E12.5). Scale bars, 1 mm.

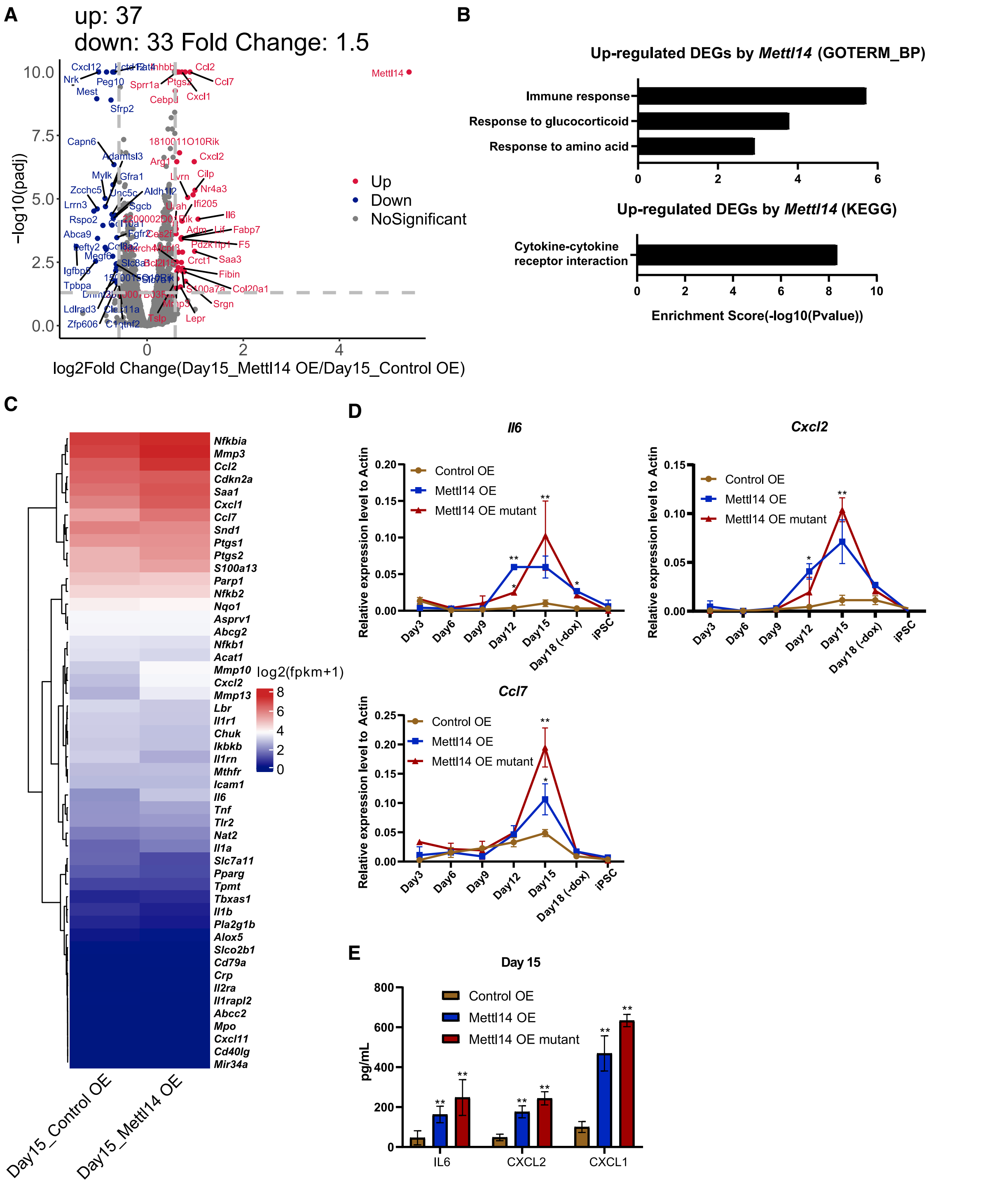

Figure 3. The expression level of SASP genes was increased after OE of *Mettl14*

(A) Volcano plot showing the DEGs representing genetic changes caused by *Mettl14* OE on day 15 of reprogramming.

(B) Gene Ontology (GO) enrichment analysis showing that the DEGs increased by *Mettl14* were mainly enriched in the immune response.

(C) Heatmap showing SASP gene clustering in the samples on reprogramming day 15.

(legend continued on next page)

culture untransfected reprogrammable MEFs (Figure 4A). Compared with medium used to culture the control group cells, conditioned medium obtained from *Mettl14* wild-type or mutant cell culture led to more untransfected reprogrammable MEFs transitioning into iPSCs (Figure 4B). Furthermore, to identify the SASP factors that facilitated the transition of somatic cells to iPSCs, we evaluated the effect of IL-6, a cytokine in the SASP, at different time points in the reprogramming process (Figure 4C). In the middle and late stages of reprogramming (days 8 and 12), IL-6 treatment significantly improved reprogramming efficiency (Figure 4D). These results suggested that SASP factors were secreted into the medium and regulated reprogramming efficiency.

It is thought that SASP factors are secreted mainly by senescent cells. To determine whether reprogrammed cells undergo senescence, we evaluated the protein expression levels of senescence markers P21 and P16 and that of components of the nuclear factor κB (NF-κB) pathway, which is upstream of the SASP factors. A western blot analysis showed that OE of the *Mettl14* or the *Mettl14* mutant resulted in significant upregulation in the expression level of P65, a major component of NF-κB complexes, as well as cyclin-dependent kinase inhibitors P16 and P21 during the late stage of reprogramming (Figure 4E).

To explore the possible relationship between *in vitro* reprogramming and senescence, we examined whether both of these processes proceeded within the same time period in different cell populations. We performed double staining for AP (indicating pluripotent colonies) and SAβG (indicating senescent cells) on day 15 of reprogramming (Mosteiro et al., 2016). We found a positive correlation between the degree of cell senescence and the number of AP^+ colonies (Figure 4F). During reprogramming, wild-type *Mettl14* and mutant *Mettl14* triggered more cells to undergo senescence and generated more iPSCs (Figure 4F).

To determine which subpopulation of cells exhibited senescence and expressed SASP genes, we sorted *Oct4*-GFP^+ and *Oct4*-GFP^- cells by fluorescence-activated cell sorting (FACS) on day 15 of reprogramming and measured the expression levels of senescence and SASP genes. The expression levels of senescence genes, such as *p16* (*Cdkn2a*) and *p21* (*Cdkn1a*), and of SASP genes, including *Il6*, *Cxcl1*, and *Ccl7*, in *Oct4*-GFP^- cells were much higher than those in *Oct4*-GFP^+ cells (Figure 4G), suggesting that the cells that had not been successfully reprogrammed (also termed non-reprogrammed [NR] cells; Guo et al., 2019) underwent senescence and secreted SASP factors.

To track which cell population expressed SASP genes, we analyzed publicly available single-cell RNA-seq data on the cell-fate continuum during somatic cell reprogramming (Guo et al., 2019). The expression patterns of *Il6*, *Cxcl1*, and *Ccl2* were consistent with those of the NR branch signature genes (*Cd34* and *klk10*) (Figure S4A) but were very different from those of reprogramming potential (RP) branch signature genes (*Sal4* and *Dppa5a*) (Figure S4A). This result suggested that SASP-producing cells were mainly in the NR branch fraction. In addition, the expression levels of SASP genes, such as *Ccl2* and *Ccl7*, in RP cells were significantly lower than those in NR cells (Figure S4B). Collectively, the findings revealed that *Mettl14* mainly enhances SASP secretion in NR cells.

To determine whether the increased efficiency of iPSC generation depends on cellular senescence or the SASP, we used small molecules to treat the reprogrammed cells on day 10 with Navitoclax (also known as ABT263) to selectively reduce the viability of senescent cells by inhibiting *Bcl-2*/*Bcl-xL*/*Bcl-w* expression (Chang et al., 2016) and BAY 11-7082, an inhibitor that blocks activation of NF-κB pathway, a master regulator of the SASP (Acosta et al., 2008; Chien et al., 2011; Freund et al., 2011; Krishnan et al., 2013; Lee et al., 2012). Both inhibitors significantly reduced the number of senescent cells, as indicated by SAβG staining, which is shown in Figure 4D, and effectively blocked the upregulation of SASP gene expression by *Mettl14* or its mutant (Figure S4C). Correspondingly, these two inhibitors blocked the activation effect of *Mettl14* on reprogramming, as indicated by the number of *Oct4*-GFP^+ and AP^+ colonies and the percentage of *Oct4*-GFP^+ cells (Figure 4H). These results suggested that the SASP is required for *Mettl14* to affect reprogramming.

In conclusion, the effect of *Mettl14* on reprogramming mainly depended on cellular senescence and transiently upregulated expression of SASP genes in NR cells during the late phase of reprogramming in an m^6A-independent manner.

DISCUSSION

We focused on the m^6A-independent function of *Mettl14* during *in vitro* reprogramming. *Mettl14* significantly upregulated the expression level of SASP genes during the late phase of reprogramming. It had been previously reported that in senescent cells, *Mettl14* regulated SASP genes in an m^6A-independent manner (Liu et al., 2021). Based our METTL14 chromatin immunoprecipitation sequencing (ChIP-seq) data, we hypothesized that *Mettl14*

(D) qRT-PCR was performed to determine the expression levels of SASP genes in wild-type and mutant *Mettl14* cells from days 3 to 18 and in iPSCs. The data are presented as the means ± SEM (n = 3); *p < 0.05, **p < 0.01 by Student's t test performed for comparison.
(E) ELISAs showing the expression levels of IL-6, CXCL2, and CXCL1 in the cell-conditioned medium on reprogramming day 15. The data are presented as the means ± SEM (n = 3); *p < 0.05, **p < 0.01 by Student's t test performed for comparison.

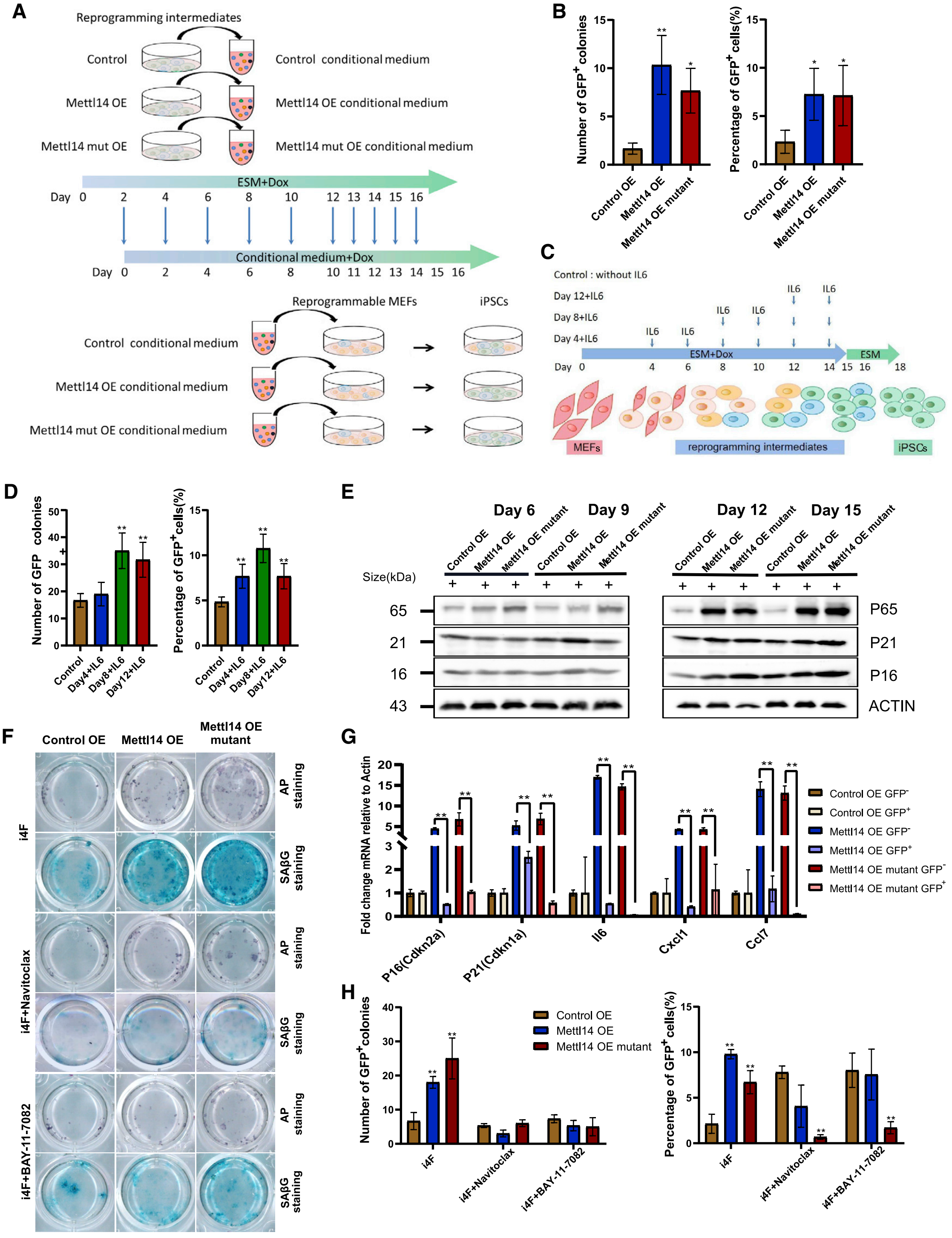

Figure 4. Reprogramming efficiency was reduced after SASP factors or senescence inhibitors were added to the culture

(A) Schematic diagram for the procedure of collection of conditional medium from the reprogramming intermediates of different groups and treatment of reprogrammable cells.

(B) Estimated reprogramming efficiency of conditioned medium from the reprogramming intermediates of different group treatments tested by the number of *Oct4*-GFP^{+} colonies formed and the percentage of *Oct4*-GFP^{+} cells. The data are presented as the means ± SEM (n = 3); *p < 0.05, **p < 0.01 by Student's t test performed for comparison.

(legend continued on next page)

functions as a transcription factor or co-activator, binds to promoter regions of SASP genes, and increases their expression to facilitate somatic cell reprogramming. Notably, our data supported the hypothesis that SASP genes facilitate reprogramming, which is consistent with their role during *in vivo* reprogramming (Mosteiro et al., 2016, 2018).

The relationship between senescence and reprogramming remains controversial. OE of OSKM genes caused both cell senescence and reprogramming. It has been previously shown that long-term OE of the inflammation-related pathway *Ink4/Arf* locus, comprising *Cdkn2a-Cdkn2b* genes that encode four potent tumor suppressors, namely $p16^{Ink4a}$, $p19^{Arfand}$, $p15^{Ink4b}$, and $p21^{Cdkn1a}$, inhibited the efficiency of *in vitro* reprogramming (Dulic et al., 2000; Hong et al., 2009; Li et al., 2009). However, in the *in vivo* reprogramming system presented in the previous study, after the KD of *Ink4/Arf* pathway components, cell senescence was sharply attenuated and cell reprogramming efficiency was reduced *in vivo* (Mosteiro et al., 2016). The most widely investigated validation factor, IL-6, activates a *Jak/Stat* target, the serine/threonine kinase gene *Pim1*, resulting in a 2-fold increase in the iPSC acquisition rate (Brady et al., 2013).

The dynamic homeostatic function of senescent cells depends on their clearance by the immune system once their beneficial function has been realized (Krizhanovsky et al., 2008; Sagiv et al., 2016). Senescence induction is required for effective cell reprogramming *in vivo*, as SASP factor production promotes reprogramming of somatic cells into iPSCs in a paracrine manner (Mosteiro et al., 2016). We analyzed our RNA-seq data and found that cytokine-cytokine receptor interactions were significantly enriched with upregulated DEGs that had been induced by *Mettl14*. It has been speculated that during reprogramming, senescent cells secrete SASP factors to promote potential reprogramming of cells, enabling them to acquire pluripotency through the paracrine process.

Therefore, we believe that short-term expression of SASP genes may have beneficial effects in different systems, such as during immune surveillance and immune clearance in senescent cells, and positive effects on reprogramming efficiency during reprogramming but that their long-term expression is detrimental to the organism.

EXPERIMENTAL PROCEDURES

The experimental procedures were including in supplementary information.

Resource availability

Accession numbers

The sequencing datasets have been deposited in the NCBI Gene Expression Omnibus (GEO) database and are accessible through GEO: GSE196475.

SUPPLEMENTAL INFORMATION

Supplemental information can be found online at https://doi.org/10.1016/j.stemcr.2022.06.012.

AUTHOR CONTRIBUTIONS

C.X. and L.W. designed and performed the experiments, performed the data analysis, led discussion, and wrote the manuscript; X.X. performed the bioinformatics analyses; C.X., Y.W., X.K., Y.Z., J. Sun, Y.D., Z.S., J. Shen, D.L., W.Y., Y.L., R.Z., Y.X., H.W., L.H., L.W. and S.G. contributed to the experimental work and discussion; and S.G. and L.W. supervised the study and contributed to writing. There is no conflict of interest in this article.

ACKNOWLEDGMENTS

We thank Professor Yawei Gao from Tongji University for providing valuable advice and Professor Jun Liu and Weide Xiao from Peking University for performing mass spectrometry to quantify m^6A. We are also grateful to our laboratory colleagues for their assistance with experiments and advice. This work was supported by the National Natural Science Foundation of China (31721003, 31801243, and 31820103009) and the key project of the Science and Technology of Shanghai Municipality (19JC1415300 and 21JC1405500).

Received: February 13, 2022
Revised: June 20, 2022
Accepted: June 21, 2022
Published: August 9, 2022

(C) Schematic diagram of IL6 treatment at different time points after the induction during reprogramming.
(D) Estimated reprogramming efficiency of IL6 treatment tested by the number of *Oct4*-GFP^+ colonies formed and the percentage of *Oct4*-GFP^+ cells. The data are presented as the means ± SEM (n = 3); *p < 0.05, **p < 0.01 by Student's t test performed for comparison.
(E) Western blot showing the expression level of NF-κB complexes regulatory subunit P65 and cyclin-dependent kinase inhibitors P16 and P21 during reprogramming. ACTIN is used as loading control.
(F) Alkaline phosphatase (AP) staining and β-galactosidase staining showing changes in the number of senescent cells and GFP^+ colonies during reprogramming before and after the treatment of Navitoclax and BAY 11-7082.
(G) qRT-PCR analysis results showing the SASP gene expression levels in *Oct4*-GFP^- and *Oct4*-GFP^+ cells. The data are presented as the means ± SEM (n = 3); *p < 0.05, **p < 0.01 by Student's t test performed for comparison.
(H) The number of *Oct4*-GFP^+ colonies was counted, and the percentage of *Oct4*-GFP^+ cells was determined by FACS 18 days after induction. Navitoclax and BAY 11-7082 were added to different experimental groups. The data are presented as the means ± SEM (n = 3); *p < 0.05, **p < 0.01 by Student's t test performed for comparison.

REFERENCES

Acosta, J.C., Banito, A., Wuestefeld, T., Georgilis, A., Janich, P., Morton, J.P., Athineos, D., Kang, T.W., Lasitschka, F., Andrulis, M., et al. (2013). A complex secretory program orchestrated by the inflammasome controls paracrine senescence. Nat. Cell Biol. *15*, 978–990.

Acosta, J.C., O'Loghlen, A., Banito, A., Guijarro, M.V., Augert, A., Raguz, S., Fumagalli, M., Da Costa, M., Brown, C., Popov, N., et al. (2008). Chemokine signaling via the CXCR2 receptor reinforces senescence. Cell *133*, 1006–1018.

Aguilo, F., Zhang, F., Sancho, A., Fidalgo, M., Di Cecilia, S., Vashisht, A., Lee, D.F., Chen, C.H., Rengasamy, M., Andino, B., et al. (2015). Coordination of m(6)A mRNA methylation and gene transcription by ZFP217 regulates pluripotency and reprogramming. Cell Stem Cell *17*, 689–704.

Alcorta, D.A., Xiong, Y., Phelps, D., Hannon, G., Beach, D., and Barrett, J.C. (1996). Involvement of the cyclin-dependent kinase inhibitor p16 (INK4a) in replicative senescence of normal human fibroblasts. Proc. Natl. Acad. Sci. USA *93*, 13742–13747.

Andriani, G.A., Almeida, V.P., Faggioli, F., Mauro, M., Tsai, W.L., Santambrogio, L., Maslov, A., Gadina, M., Campisi, J., Vijg, J., and Montagna, C. (2016). Whole Chromosome Instability induces senescence and promotes SASP. Sci. Rep. *6*, 35218.

Batista, P.J., Molinie, B., Wang, J., Qu, K., Zhang, J., Li, L., Bouley, D.M., Lujan, E., Haddad, B., Daneshvar, K., et al. (2014). m(6)A RNA modification controls cell fate transition in mammalian embryonic stem cells. Cell Stem Cell *15*, 707–719.

Brady, J.J., Li, M., Suthram, S., Jiang, H., Wong, W.H., and Blau, H.M. (2013). Early role for IL-6 signalling during generation of induced pluripotent stem cells revealed by heterokaryon RNA-Seq. Nat. Cell Biol. *15*, 1244–1252.

Carey, B.W., Markoulaki, S., Beard, C., Hanna, J., and Jaenisch, R. (2010). Single-gene transgenic mouse strains for reprogramming adult somatic cells. Nat. Methods *7*, 56–59.

Chang, J., Wang, Y., Shao, L., Laberge, R.M., Demaria, M., Campisi, J., Janakiraman, K., Sharpless, N.E., Ding, S., Feng, W., et al. (2016). Clearance of senescent cells by ABT263 rejuvenates aged hematopoietic stem cells in mice. Nat. Med. *22*, 78–83.

Chen, T., Hao, Y.J., Zhang, Y., Li, M.M., Wang, M., Han, W., Wu, Y., Lv, Y., Hao, J., Wang, L., et al. (2015). m(6)A RNA methylation is regulated by microRNAs and promotes reprogramming to pluripotency. Cell Stem Cell *16*, 289–301.

Chien, Y., Scuoppo, C., Wang, X., Fang, X., Balgley, B., Bolden, J.E., Premsrirut, P., Luo, W., Chicas, A., Lee, C.S., et al. (2011). Control of the senescence-associated secretory phenotype by NF-kappaB promotes senescence and enhances chemosensitivity. Genes Dev. *25*, 2125–2136.

Dulić, V., Beney, G.E., Frebourg, G., Drullinger, L.F., and Stein, G.H. (2000). Uncoupling between phenotypic senescence and cell cycle arrest in aging p21-deficient fibroblasts. Mol. Cell Biol. *20*, 6741–6754.

Fitzner, B., Müller, S., Walther, M., Fischer, M., Engelmann, R., Müller-Hilke, B., Pützer, B.M., Kreutzer, M., Nizze, H., and Jaster, R. (2012). Senescence determines the fate of activated rat pancreatic stellate cells. J. Cell Mol. Med. *16*, 2620–2630.

Freund, A., Patil, C.K., and Campisi, J. (2011). p38MAPK is a novel DNA damage response-independent regulator of the senescence-associated secretory phenotype. EMBO J. *30*, 1536–1548.

Geula, S., Moshitch-Moshkovitz, S., Dominissini, D., Mansour, A.A., Kol, N., Salmon-Divon, M., Hershkovitz, V., Peer, E., Mor, N., Manor, Y.S., et al. (2015). Stem cells. m6A mRNA methylation facilitates resolution of naive pluripotency toward differentiation. Science *347*, 1002–1006.

Guo, L., Lin, L., Wang, X., Gao, M., Cao, S., Mai, Y., Wu, F., Kuang, J., Liu, H., Yang, J., et al. (2019). Resolving cell fate decisions during somatic cell reprogramming by single-cell RNA-Seq. Mol. Cell *73*, 815–829.e7.

Hong, H., Takahashi, K., Ichisaka, T., Aoi, T., Kanagawa, O., Nakagawa, M., Okita, K., and Yamanaka, S. (2009). Suppression of induced pluripotent stem cell generation by the p53-p21 pathway. Nature *460*, 1132–1135.

Kang, L., and Gao, S. (2012). Pluripotency of induced pluripotent stem cells. J. Anim. Sci. Biotechnol. *3*, 5.

Kang, L., Wang, J., Zhang, Y., Kou, Z., and Gao, S. (2009). iPS cells can support full-term development of tetraploid blastocyst-complemented embryos. Cell Stem Cell *5*, 135–138.

Krishnan, N., Bencze, G., Cohen, P., and Tonks, N.K. (2013). The anti-inflammatory compound BAY-11-7082 is a potent inhibitor of protein tyrosine phosphatases. FEBS J. *280*, 2830–2841.

Krizhanovsky, V., Yon, M., Dickins, R.A., Hearn, S., Simon, J., Miething, C., Yee, H., Zender, L., and Lowe, S.W. (2008). Senescence of activated stellate cells limits liver fibrosis. Cell *134*, 657–667.

Le, R., Kou, Z., Jiang, Y., Li, M., Huang, B., Liu, W., Li, H., Kou, X., He, W., Rudolph, K.L., et al. (2014). Enhanced telomere rejuvenation in pluripotent cells reprogrammed via nuclear transfer relative to induced pluripotent stem cells. Cell Stem Cell *14*, 27–39.

Lee, J., Rhee, M.H., Kim, E., and Cho, J.Y. (2012). BAY 11-7082 is a broad-spectrum inhibitor with anti-inflammatory activity against multiple targets. Mediators Inflamm. *2012*, 416036.

Li, H., Collado, M., Villasante, A., Strati, K., Ortega, S., Canamero, M., Blasco, M.A., and Serrano, M. (2009). The Ink4/Arf locus is a barrier for iPS cell reprogramming. Nature *460*, 1136–1139.

Liu, P., Li, F., Lin, J., Fukumoto, T., Nacarelli, T., Hao, X., Kossenkov, A.V., Simon, M.C., and Zhang, R. (2021). m(6)A-independent genome-wide METTL3 and METTL14 redistribution drives the senescence-associated secretory phenotype. Nat. Cell Biol. *23*, 355–365.

Lopes-Paciencia, S., Saint-Germain, E., Rowell, M.C., Ruiz, A.F., Kalegari, P., and Ferbeyre, G. (2019). The senescence-associated secretory phenotype and its regulation. Cytokine *117*, 15–22.

Marcheggiani, F., Kordes, S., Cirilli, I., Orlando, P., Silvestri, S., Vogelsang, A., Moller, N., Blatt, T., Weise, J.M., Damiani, E., et al. (2021). Anti-ageing effects of ubiquinone and ubiquinol in a senescence model of human dermal fibroblasts. Free Radic. Biol. Med. *165*, 282–288.

Mosteiro, L., Pantoja, C., Alcazar, N., Marion, R.M., Chondronasiou, D., Rovira, M., Fernandez-Marcos, P.J., Munoz-Martin, M., Blanco-Aparicio, C., Pastor, J., et al. (2016). Tissue damage and

senescence provide critical signals for cellular reprogramming in vivo. Science *354*.

Mosteiro, L., Pantoja, C., de Martino, A., and Serrano, M. (2018). Senescence promotes in vivo reprogramming through p16(INK)(4a) and IL-6. Aging Cell *17*.

Sagiv, A., Burton, D.G., Moshayev, Z., Vadai, E., Wensveen, F., Ben-Dor, S., Golani, O., Polic, B., and Krizhanovsky, V. (2016). NKG2D ligands mediate immunosurveillance of senescent cells. Aging (Albany NY) *8*, 328–344.

Suvakov, S., Cubro, H., White, W.M., Butler Tobah, Y.S., Weissgerber, T.L., Jordan, K.L., Zhu, X.Y., Woollard, J.R., Chebib, F.T., Milic, N.M., et al. (2019). Targeting senescence improves angiogenic potential of adipose-derived mesenchymal stem cells in patients with preeclampsia. Biol. Sex Differ. *10*, 49.

Takahashi, K., and Yamanaka, S. (2006). Induction of pluripotent stem cells from mouse embryonic and adult fibroblast cultures by defined factors. Cell *126*, 663–676.

Wang, Y., Li, Y., Toth, J.I., Petroski, M.D., Zhang, Z., and Zhao, J.C. (2014). N6-methyladenosine modification destabilizes developmental regulators in embryonic stem cells. Nat. Cell Biol. *16*, 191–198.

Wang, X., Feng, J., Xue, Y., Guan, Z., Zhang, D., Liu, Z., Gong, Z., Wang, Q., Huang, J., Tang, C., et al. (2016). Structural basis of N(6)-adenosine methylation by the METTL3-METTL14 complex. Nature *534*, 575–578.

Wen, J., Lv, R., Ma, H., Shen, H., He, C., Wang, J., Jiao, F., Liu, H., Yang, P., Tan, L., et al. (2018). Zc3h13 Regulates nuclear RNA m(6)A methylation and mouse embryonic stem cell Self-Renewal. Mol. Cell *69*, 1028–1038.e6.

Wu, L., Wu, Y., Peng, B., Hou, Z., Dong, Y., Chen, K., Guo, M., Li, H., Chen, X., Kou, X., et al. (2017). Oocyte-specific Homeobox 1, Obox1, facilitates reprogramming by promoting mesenchymal-to-epithelial transition and mitigating cell hyperproliferation. Stem Cell Rep. *9*, 1692–1705.

Wu, L., He, S., Ye, W., Shen, J., Zhao, K., Zhang, Y., Zhang, R., Wei, J., Cao, S., Chen, K., et al. (2021). Surf4 facilitates reprogramming by activating the cellular response to endoplasmic reticulum stress. Cell Prolif. *54*, e13133.

You, K., Parikh, P., Khandalavala, K., Wicher, S.A., Manlove, L., Yang, B., Roesler, A., Roos, B.B., Teske, J.J., Britt, R.D., Jr., et al. (2019). Moderate hyperoxia induces senescence in developing human lung fibroblasts. Am. J. Physiol. Lung Cell Mol. Physiol. *317*, L525–L536.

Yun, M.H., Davaapil, H., and Brockes, J.P. (2015). Recurrent turnover of senescent cells during regeneration of a complex structure. Elife *4*.

Zaccara, S., Ries, R.J., and Jaffrey, S.R. (2019). Reading, writing and erasing mRNA methylation. Nat. Rev. Mol. Cell Biol. *20*, 608–624.

Zhao, Y., Shi, Y., Shen, H., and Xie, W. (2020). m(6)A-binding proteins: the emerging crucial performers in epigenetics. J. Hematol. Oncol. *13*, 35.

Stem Cell Reports, Volume *17*

Supplemental Information

Mettl14-driven senescence-associated secretory phenotype facilitates somatic cell reprogramming

Chenxiang Xi, Jiatong Sun, Xiaocui Xu, You Wu, Xiaochen Kou, Yanhong Zhao, Jiacheng Shen, Yu Dong, Kang Chen, Zhongqu Su, Dan Liu, Wen Ye, Yingdong Liu, Ran Zhang, Yiliang Xu, Hong Wang, Lujiang Hao, Li Wu, and Shaorong Gao

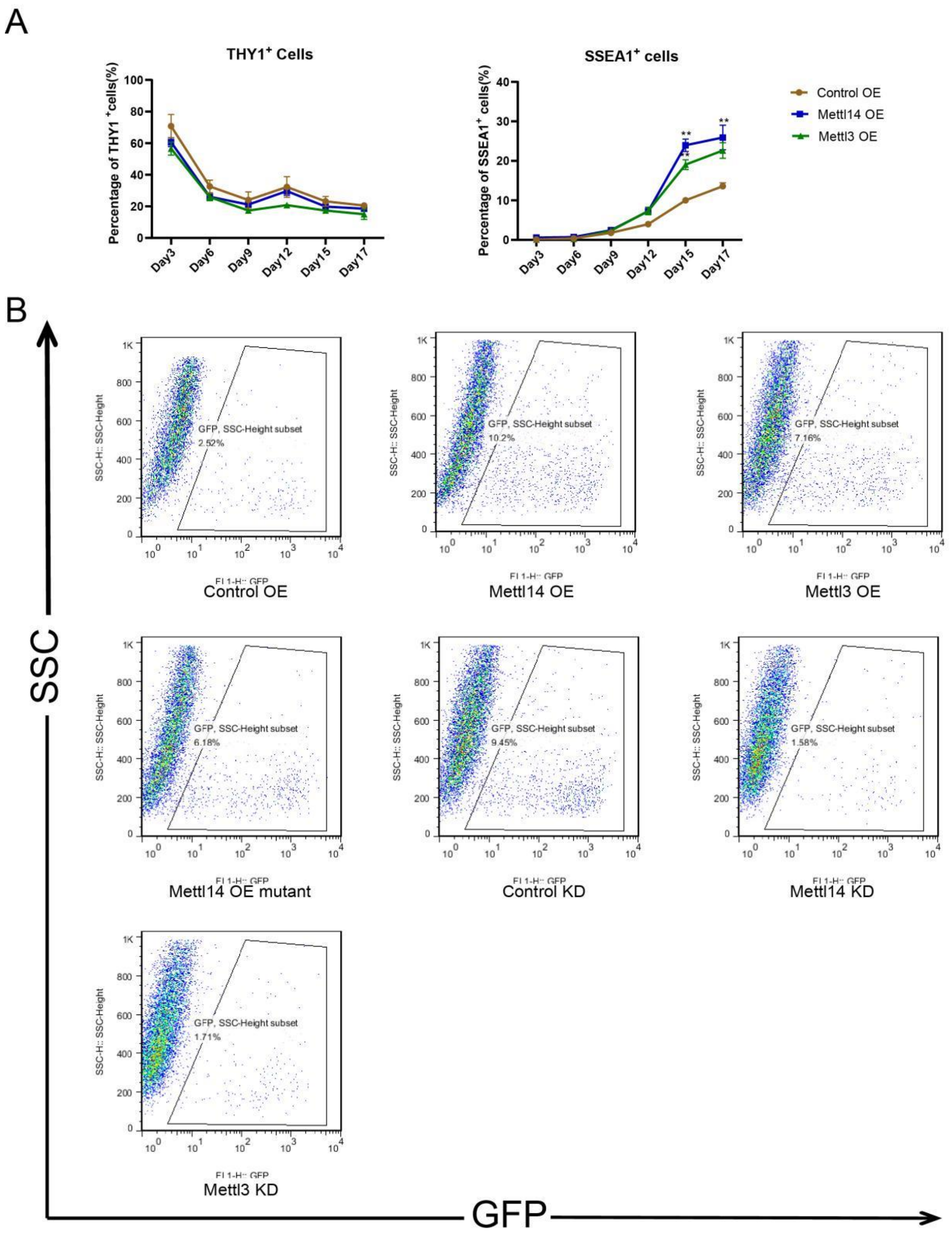

Figure S1. *Mettl14* can facilitate reprogramming in an m^{6}A-independent manner. Related to Figure 1.

(A) Kinetic changes of percentage of THY1$^+$ and SSEA1$^+$ population at indicated time points during reprogramming by FACS analysis. Data are represented as the mean ± SEM (n = 3); * $p < 0.05$, ** $p < 0.01$ by Student's *t*-test for comparison.

(B) FACS analysis showed that the proportion of *Oct4*-GFP$^+$ cells in each experimental group on Day 18 after induction.

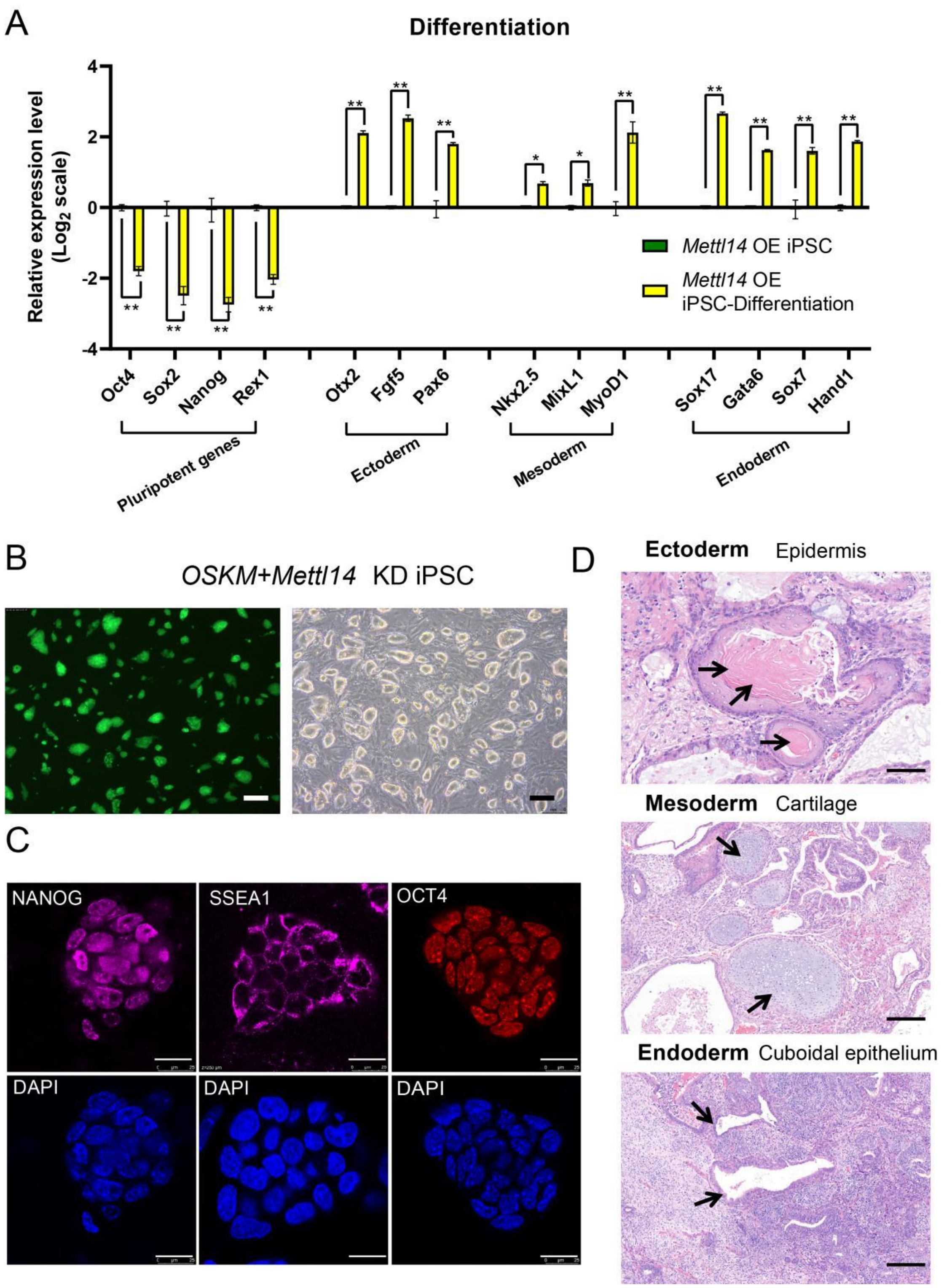

Figure S2. iPSC lines with OSKM+*Mettl14* OE or KD exhibit pluripotency. Related to Figure 2.

(A) qRT-PCR analysis shows iPSCs with OSKM+*Mettl14* OE line's embryoid bodies can subsequently differentiate and express the three embryonic marker genes. Data are represented as the mean ± SEM (n = 3); * $p < 0.05$, ** $p < 0.01$ by Student's *t*-test for comparison.

(B) Morphology of the iPSCs with OSKM+*Mettl14* KD lines. Scale bars, 200 µm.

(C) Immunostaining analyses for the expression of pluripotent marker genes NANOG (purple), SSEA1 (purple) and OCT4 (red), and in the iPSCs with OSKM+*Mettl14* KD lines. Nuclear staining by DAPI (blue). Scale bars, 25 µm.

(D) Haematoxylin and eosin (H&E) staining of teratomas generated by the iPSCs with OSKM+*Mettl14* KD. Scale bars, 100 µm.

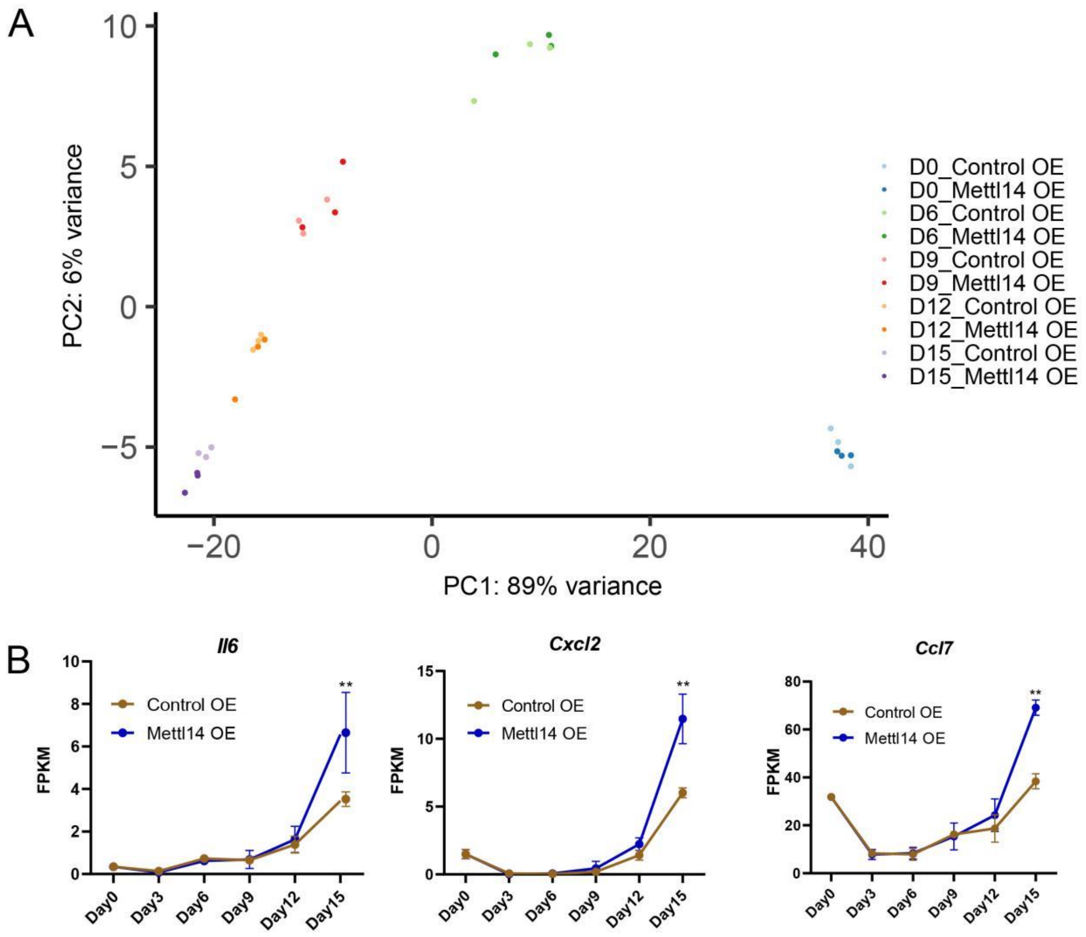

Figure S3. The expression level of SASP gene was increased after overexpression of *Mettl14.* Related to Figure 3.

(A) PCA analysis showing changes at various time points during reprogramming**.**

(B) FPKM shows changes in differential genes during reprogramming. Data are represented as the mean ± SEM (n = 3); * $p < 0.05$, ** $p < 0.01$ by Student's *t*-test for comparison.

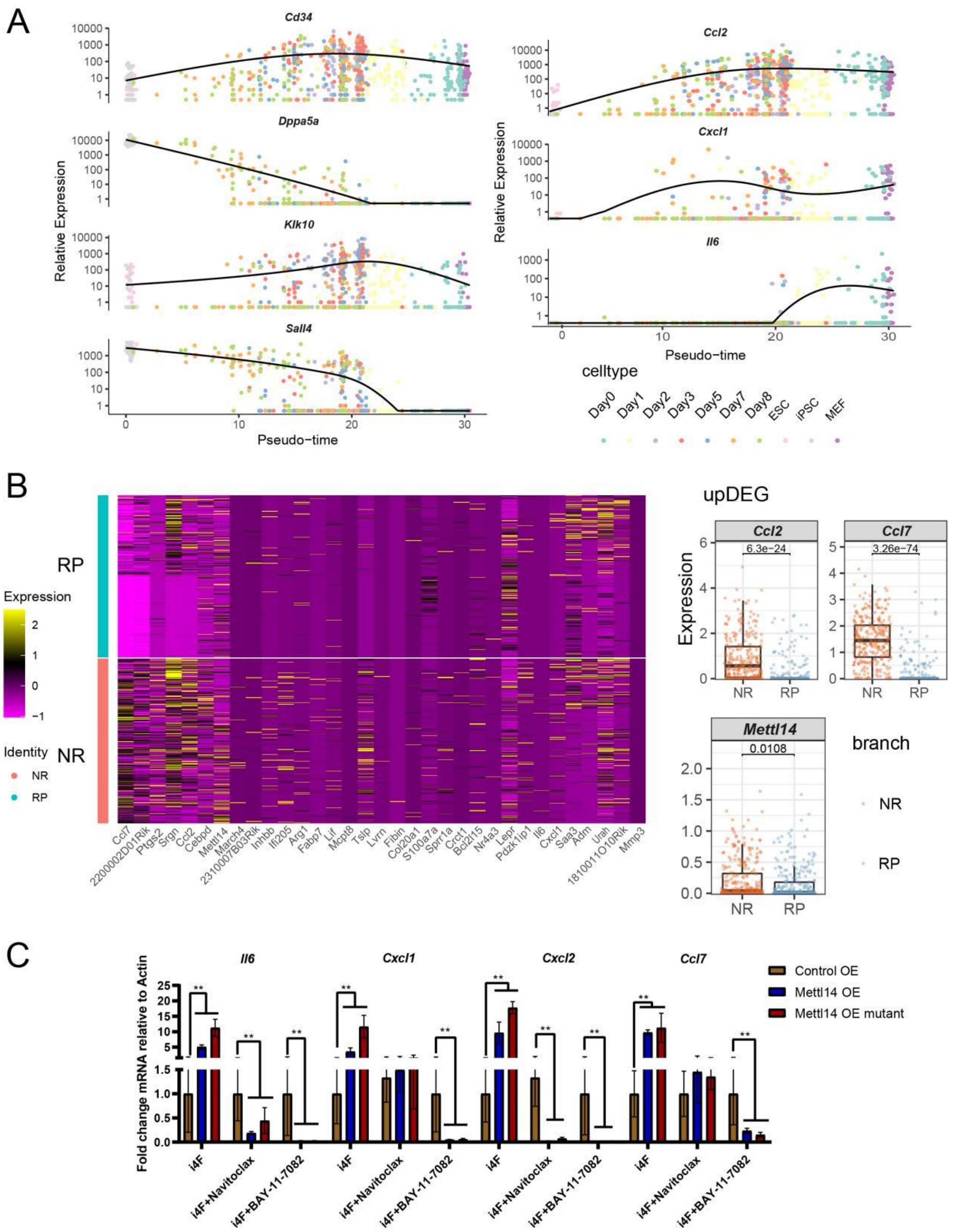

Figure S4. Reprogramming efficiency was reduced after treatment of SASP or senescence inhibitor*.* Related to Figure 4.

(A) Expression of the non-reprogramming (NR) branch signature genes (*Cd34* and *Klk10*) and reprogramming potential (RP) branch signature genes (*Sal4* and *Dppa5a*) along the path of the pseudotime.

(B) Identification of the differentially expressed genes between NR and RP branches using Beta-Poisson model for single-cell RNA-seq data analyses (BPSC) with adjusted p < 0.05 by Student's *t*-test for comparison..

(C) qRT-PCR analysis shows SASP gene expression in the reprogramming system with

the addition of inhibitors, shows the proportion of Fold change in these genes compared to control, represented relative to expression in *β-Actin*. Data are represented as the mean ± SEM (n = 3); * $p < 0.05$, ** $p < 0.01$ by Student's *t*-test for comparison.

Table S1. Primer Sequences Used in this Paper. Related to Figure 2/S2/3/S3/4/S4.

Primer Name	Reverse Primer	Forward Primer
Oct4	CGAAGCGACAGATGGTGGTC	AGAGGATCACCTTGGGGTACA
Sox2	TGCTTTGTCCGTATCCAGTGC	AGCAATGGTTCTTATGTTGGACG
Nanog	GCAAGAATAGTTCTCGGGATGAA	CACAGTTTGCCTAGTTCTGAGG
Rex1	TCTGGGTTGTACGGGTCATAG	ATGCTACGTTCTACCGGCTTC
Otx2	GCCCTAGTAAATGTCGTCCTCTC	TATCTAAAGCAACCGCCTTACG
Fgf5	GAAGAAAACGTCGCGCTACT	GAAGCGTCTCACTCCCGAAG
Pax6	GAGTCGCCACTCTTGGCTTA	GTTGTGTGAGTAAAATTCTGGGC
Nkx2-5	CTGTCGCTTGCACTTGTAGC	GACAAAGCCGAGACGGATGG
MixL1	TCCCAGGAGTCCAACTTTGAG	ACTGAAGCTAGGTGTTTGAAGC
MyoD1	TCGAAACACGGGTCATCATAGA	CGGGACATAGACTTGACAGGC
Sox17	CCACCTCGCCTTTCACCTTTA	GATGCGGGATACGCCAGTG
Gata6	GTGGTCGCTTGTGTAGAAGGA	TTGCTCCGGTAACAGCAGTG
Sox7	CGTGTTCTGGTCACGAGAGA	ATGCTGGGAAAGTCATGGAAG
Hand1	GCATCGGGACCATAGGCAG	GGCAGCTACGCACATCATCA
Cxcl1	AACCAAGGGAGCTTCAGGGTCA	TCCAGAGCTTGAAGGTGTTGCC
Cxcl2	GGCTTCAGGGTCAAGGCAAACT	CATCCAGAGCTTGAGTGTGACG
Ccl2	GTCTGGACCCATTCCTTCTTGG	GCTACAAGAGGATCACCAGCAG
Ccl7	ATAGCCTCCTCGACCCACTTCT	CAGAAGGATCACCAGTAGTCGG
Il6	CTGCAAGTGCATCATCGTTGTTC	TACCACTTCACAAGTCGGAGGC
S100a7a	CTGGAGATGGTAGTCCTTCACC	GATAGTGTGCCTCGCTTCATGG
Calca	CTCAGATTCCCACACCGCTTAG	GCACTGGTGCAGGACTATATGC
Trp53	GAGGCCGGCTCTGAGTATACC	GTCCCAGAAGGTTCCCACTGGA
Cdkn1a	CCAATCTGCGCTTGGAGTGATAG	TCGCTGTCTTGCACTCTGGTGT
Cdkn2a	CGAATCTGCACCGTAGTTGAGC	TGTTGAGGCTAGAGAGGATCTTG
Rela	GGTCTCATAGGTCCTTTTGCGC	TCCTGTTCGAGTCTCCATGCAG
Mettl3	TGAGAGGTGGTGTAGCAACTT	CTGGGCACTTGGATTTAAGGAA
Mettl14	AGGTCCAATCCTTCCCCAGAA	GACTGGCATCACTGCGAATGA

Table S2. FPKM of genes in volcano and heat maps after overexpression of *Mettl14* on Day 15 of reprogramming in RNA-seq Data.

Gene Name	Day15 Control-1	Day15 Control-2	Day15 Control-3	Day15 Mettl14OE-1	Day15 Mettl14OE-2	Day15 Mettl14OE-3
Il6	3.10071	3.42969	3.7825	4.64235	8.43489	6.61467
Cilp	1.0128	1.50932	1.58887	3.11203	2.65677	2.40388
Saa3	8.63937	9.56221	13.9514	20.1873	29.933	11.9028
Cxcl2	6.42135	5.6963	5.94721	9.44787	12.9886	11.9734
Nr4a3	1.14686	1.78633	2.09913	4.05531	3.63952	3.6047
Ccl7	38.0989	41.6011	35.4044	72.7539	67.5895	66.9442

Lvrn	1.87122	2.45833	2.0439	3.84444	4.35767	3.27718
Srgn	73.2929	79.4373	48.5089	118.761	110.202	109.196
Ccl2	100.858	87.6044	86.9499	151.945	162.251	144.982
Col20a1	0.253553	0.424448	0.357017	0.549747	0.676647	0.550783
Fibin	2.09973	2.48539	3.49579	4.0921	4.15271	4.8317
Pdzk1ip1	11.6125	13.1095	8.74607	21.689	15.8428	16.597
S100a7a	4.316	3.54186	4.98286	8.49985	7.75266	7.92842
Ifi205	6.91898	7.39863	7.36023	10.825	12.0357	12.1769
Cxcl1	61.6357	59.0066	56.2672	97.6691	85.0323	99.7257
Lif	2.82955	2.55727	2.63662	3.25625	4.94839	4.42052
Crct1	17.9904	25.0129	15.2178	37.5422	29.7866	25.2319
F5	1.02493	1.19734	1.28256	2.24806	1.73628	1.55523
Fabp7	14.4991	12.3987	12.6412	18.2676	23.5247	19.2173
Lepr	0.941787	1.07526	1.106	1.13498	2.0702	2.02172
1810011O1	18.4608	20.5147	19.0956	31.4921	33.2224	25.2479
Inhbb	27.8833	28.4317	31.4826	47.3002	47.7918	38.4
Mcpt8	14.1991	10.7608	13.802	22.6779	18.5148	15.1304
Bcl2l15	6.09294	6.00122	5.60685	9.11008	8.56783	8.19252
Ptgs2	29.4949	30.585	35.2759	45.921	47.9811	45.6273
Tslp	6.88578	5.68467	9.0653	9.44746	11.7001	10.5428
2310007B0	3.68851	2.74692	2.72075	4.07112	4.78983	4.83833
Sprr1a	101.242	96.635	104.68	160.959	151.63	129.267
Arg1	16.4418	19.2019	17.5435	26.9708	27.3734	24.8046
Mmp3	135.827	97.636	134.692	189.83	211.784	138.147
Adm	11.7006	11.0716	14.7973	21.057	17.6963	15.5685
Ces2f	11.1061	12.7747	9.26462	16.4765	17.8925	13.4787
Urah	21.6548	22.2704	24.2253	31.4051	33.0631	33.3812
2200002D0	88.0926	89.1785	70.3239	126.75	106.258	132.082
Cebpd	20.8436	22.8618	24.0382	33.7725	32.3735	31.3504

SUPPLEMENTAL EXPERIMENTAL PROCEDURES

Mice

Oct4-GFP$^+$ (OG2) mice (Jackson laboratory code 004654) were crossed with R26rtTA; Col1a1-4F2A mice (Jackson laboratory code 011004) to obtain Rosa26-M2rtTA; *Col1a1*-4F2A; *Oct4*-GFP mice. The pathogen-free mice were housed in SPF-level mouse houses at Tongji University. All of our animal research methods were in accordance with Tongji University guidelines for the use of laboratory animals.

Cell culture for MEFs, feeders and iPSCs

Mouse embryonic fibroblasts (MEFs) for iPSC induction were derived from 12.5-13.5 days embryos (Embryos were derived from the above transgenic mice), and MEFs were cultured in Dulbecco's Modified Eagle Medium (DMEM) (Gibco C11960500BT) supplemented with 1 mM L-glutamine (EmbryoMax TMS-002-C), and 10% (vol/vol) fetal bovine serum (FBS) (Gibco 10270-106). Feeder was obtained by treating the obtained MEFs with mitomycin C for three hours to render them incompetent for proliferation. iPSCs was cultured on feeder in embryonic stem cell medium (ESM), a medium of DMEM containing 1% nonessential amino acid (NEAA), stock (Merck Millipore TMS-001-C), 1 mM L-glutamine (Merck Millipore TMS-002-C), 15% (v/v)

FBS (Gibco 16000-44), 0.1 mM *β*-mercaptoethanol (Merck Millipore ES-007-E) and 1000 U/ml leukaemia inhibitory factor (LIF) (Merck Millipore ESGRO 1107).

iPSCs derivation

The HEK293T cells were transfect with overexpression or knockout plasmids, lentivirus packaging plasmid psPAX2 and pMD2G using VigoFect transfection reagent (Vigorous Biotechnology), and replaced fresh ESM 10 hours after transfection. After 48 hours of transfection, the virus-containing supernatant was collected and filtered using a 0.45 μm filter (Millex SLHV033RB). For overexpression, the starting MEFs were seeded in 12-well plates at a density of 0.6-0.8×10^4 cells/well, and for knockdown, the starting MEFs at a density of 1.2-1.3×10^4 cells/well. Cells were then infected with collected virus-containing cultures for 8-12 hours. Infected MEFs were cultured in ESM supplemented with 1 μg/mL Dox for 16-18 days. After clonal morphology formation, culture was continued in ESM without Dox for 2-3 days and individual clonal colonies were picked to establish iPS cell lines.

The iPS colonies will appear after two weeks of Dox treatment, and counted under the microscope at the indicated time points. The number of *Oct4*-GFP$^+$ colonies was shown as the number in each well in a 12-well plate.

Flow cytometry analysis

Induced cells at indicated time points were washed once with DPBS, digested with trypsin and EDTA (TE) solution and neutralized with serum-containing medium. The cells were centrifuged and resuspended with FACS buffer (PBS+0.1% BSA) and filtered for FACS assay. If antibody incubation is required, the cells were resuspended at appropriate density with FACS buffer. The antibodies were added according to the antibody instruction, and incubate for 30 minutes on ice protecting from light. The cells then washed using FACS buffer once and filtered. Flow analysis instrument used in this experiment was provided by FACS Calibur (BD, CA 95131), and the sorting flow analyzer was provided by FACS Aria II (BD).

Cell growth curve

MEFs were seeded in 12-well plates and changed fresh ESM with Dox every other day. The cells were harvested every three days until the end of reprogramming induction by withdrawal of Dox. Three replicates were containing for each sample at each time point. The cells were counted by hemocytometer.

RNA isolate and real time PCR

The cell samples were lysed with RNAiso Plus. Total RNA was extracted with TRNzol Universal Reagent (Tiangen), reverse transcribed to cDNA using 5× All-In-One TE MasterMix (Abm Cat. G490), and reverse transcribed using Eppendorf AG (223331). qRT-PCR was performed in three replicates per sample, using the ΔCT or ΔΔCT method to normalize the data, and the internal reference was *β-actin*.

Alkaline phosphatase (AP) staining

At the end of reprogramming, the cells in the 12-well plate were washed once with DPBS, then fixed in 10% formaldehyde for 5 minutes at room temperature, and washed three times

with DPBS. The fixed cells were stained using the Beyotime BCIP/NBT alkaline Phosphatase Color Development Kit (Cat. No. C3206). The staining solution is incubated for 30 minutes at room temperature and protected from light, and the staining is stopped by washing twice with DPBS.

Senescence *β*-Galactosidase (*β*-Gal) Staining

The cells in the cell culture plate were washed once with DPBS. The Senescence *β*-Galactosidase Staining Kit (Beyotime Cat.No.C0602) was used for staining. Briefly, the cells were fixed by *β*-galactosidase staining fixative at room temperature for 15 minutes. Then the cell fixative was aspirated and was washed with PBS 3 times and stained with staining working solution at 37 °C overnight. The stain was removed at the end of staining, and the cells were washed once with DPBS to abort the staining, and store at 4 °C. Taking photos of stained cells using Microsystems CMS GmbH (Leica, D-35578 Wetzlar)

Immunofluorescence (IF) staining

The iPSCs was planted in a 12-well plate with slides, and the colonies were obtained at an appropriate size. The cells were fixed using 4% paraformaldehyde (PFA) for 1 hour at room temperature, rinsed in PBS and permeabilized with 0.3% Trition X-100 in DPBS for 15 minutes at room temperature. The cells were washed with DPBS and blocked with blocking solution (2.5% BSA in DPBS) at room temperature for 1 hour. The samples were incubated with primary antibody OCT4 (1:1000, Santa Cruz, SC-5279), NANOG (1:1000, Cosmo Bio, RCAB001P) diluted in blocking solution incubate for 2 hours at room temperature, then washed three times with DPBS, and incubated secondary antibody Alexa Fluor 594 donkey anti-mouse IgG (Thermo Fisher, A21203), or Alexa Fluor 594 donkey anti-rabbit IgG (Thermo Fisher, A21207). DAPI (1 µg/mL, Merck Millipore) diluted in DPBS was used to labeled DNA for 5-10 min at room temperature. The samples were imaged using Leica Microsystems (Type: TCS SP8) confocal microscope.

Embryoid body (EB) differentiation

The iPSCs were trypsinized and plated onto tissue culture plates for 30 minutes to deplete feeder cells. The supernatant cells were collected and incubated for two days with 5×10^4 cell suspensions per drop and transferred to ultra-low cluster plates (Costar). The cells were cultured in ESM without LIF. EBs was collected after 5-10 days and seeded in gelatin-coated tissue culture dishes for 14 days. Total RNA of the cells was then extracted and the analyzed of marker genes in the three germ layers was detected by qRT-PCR.

Teratoma formation

The iPSCs were trypsinized and resuspended with DPBS at $2\text{-}3 \times 10^6$ and injected subcutaneously in the groin of SCID mice. Three to four weeks later, tumor-like growths were seen at the injection site. Mice were dislocated and executed at the neck. The teratomas were stripped, placed in 10% formaldehyde solution, fixed overnight at room temperature, stained for hematoxylin-eosin staining, and the sections were observed under a microscope and identify the iconic tissue structures of the skin epithelium (ectoderm), cartilage (mesoderm) and cuboidal epithelium (endoderm), and take photos to record.

RNA-sequencing and data processing

Collect RNA samples during reprogramming different time points, three biological replicates for each sample. Total RNA was isolated using QIAGEN RNasy Kit (14104, Germantown, US). The RNA sequencing libraries were generated using a KAPA Stranded RNA-Seq Kit Illumina platform (KK8440, Wilmington, US). Paired- end 150-bp sequencing was further performed on a HiSeq 2500 (Illumina) at Berry Genomics Corporation.

RNA-seq data were first subjected to Trim_galore (version 0.6.4) for adaptor trimming as well as quality control with the parameters --paired -j 7 --basename. The trimmed paired-end reads were then aligned to mm9 reference genome with random chromosome cleaned by STAR (version 2.7.3a) under the parameters --runThreadN 30 --runMode alignReads --outSAMtype BAM SortedByCoordinate --outSAMstrandField intronMotif. The expression of genes was quantified as FPKM by Cufflinks (version 2.2.1). For the downstream data analyses, FPKM values were averaged for each gene between replicates. The RefSeq gene annotation files were downloaded from UCSC. For genes with multiple isoforms, the longest transcripts were selected. The R package DESeq2 (version 1.26.0) were used for gene differential expression analysis. Fold change > 1.5 and FDR < 0.05 were used as cutoff for down-regulated and up-regulated genes. To perform PCA analysis, read counts per gene were summarized by the featureCounts function from Subread package. Then the raw counts were subjected to Variance Stabilizing Transformation by vst function from DESeq2 package, and then PCA values were calculated by plotPCA function from DESeq2 package.

Gene Ontology (GO) enrichment was analyzed by Gene Ontology biological process (GO_BP) and Kyoto Encyclopedia of Genes and Genomes (KEGG) in the Database for Visualization, Annotation and Integrated Discovery (DAVID) web- accessible tool. Gene ontology terms for each function cluster were summarized to a representative term, and p-values were plotted to show the significance.

ELISA

The kit used for the experiment was ABclonal ELISA Kits (Cat.No:RK00038), Specific experimental procedures refer to the instructions.

IL6 cytokines

Add 0.05ng/ml Recombinant Murine IL6 (PEPROTECH, Catalog #216-16) to the cells at different periods of the reprogramming process to explore the effect of IL6 on the reprogramming process, and count the reprogramming efficiency of each experimental group after completing the induction.

Conditional medium induce

The conditional medium of each experimental group during reprogramming was collected. The collected medium was added to the untransfected reprogrammable MEF cells (The induction time of transfected reprogrammable MEF cells(conditional medium-providing group) was two days earlier than that of non-transfected reprogrammable MEF cells, and 1 μg/mL Dox supplementation to non-transfected MEF cells was required after the addition of cell conditional medium), and the cells were cultured until the conditional medium-providing group

completed the reprogramming induction process, and the untransfected reprogrammable MEF cells were assayed for reprogramming efficiency.

Western blot analysis

Cells were washed once with DPBS, digested with trypsin and EDTA (TE) solution, lysed with a cell lysis solution containing 20 mM Tris (pH 7.5), 150 mM NaCl, 1% Triton X-100, and various protein inhibitors such as sodium pyrophosphate, *β*-glycerophosphate, EDTA, leupeptin solution. Cells were lysed by adding 1 mL of lysis buffer per 10^7 cells on ice for half an hour and then ultrasonicated. Protein concentration was determined with Enhances BCA Protein Assay Kit (Beyotime, Cat No.P0010). The samples were boiled to 100 °C for 10-15 minutes in loading buffer (EpiZyme, LT101S) with 2% *β*-mercaptoethanol (Amersham, CT). Western blot experiment (Protein electrophoresis, protein transfer, antibody containment) was performed according to Abcam Western blot protocol. Primary antibody use: anti-METTL14 (Sigma, Cat. No. HPA038002; 2 µg for immunoprecipitation, 1:1,000 for western blots), anti-NF-κB P65 (Cell Signaling, Cat. No. 8242; 1:1,000 for western blots), anti-P16 (Santa Cruz Biotechnology, Cat. No. sc-56330; 1:1,000 for western blots), anti-P21 (Abcam, Cat. No. 7960; 1:1,000 for western blots), anti-*β-Actin* (Merck, Cat. No. A1978, 1:1,000 for western blots). The secondary antibody used for Western blot were ECL peroxidase-labelled sheep anti-mouse antibody (GE Healthcare, NA931V) or HRP-labelled goat anti-rabbit antibody (Beyotime, A0208).

Single cell RNA-seq data analysis

Raw read counts of single cell RNA-seq data for samples of iPSC generation process were downloaded from GSE103221 (Guo et al., 2019). The raw read counts were then converted to HDF5 format by the count-to-h5 function from MAESTRO package (version 1.3.1). The expression matrices were then loaded into R (version 4.0.5) with Seurat (version 4.0.3). The Seurat object was then subjected to pseudotime analysis by Monocle (v.2.18.0). The gene pseudotime plots were generated by plot_genes_in_pseudotime function from Monocle package, and the heatmaps were generated by DoHeatmap function from Seurat package.

Statistical Analysis

Results were represented as the mean ± SEM of independent experiments. Significance was determined with Student's *t* tests.

Supplemental reference

Guo, L., Lin, L., Wang, X., Gao, M., Cao, S., Mai, Y., Wu, F., Kuang, J., Liu, H., Yang, J.*, et al.* (2019). Resolving Cell Fate Decisions during Somatic Cell Reprogramming by Single-Cell RNA-Seq. Mol Cell *73*, 815-829 e817.
